# Metformin and N-terminal pro B-type natriuretic peptide in type 2 diabetes patients, a post-hoc analysis of a randomized controlled trial

**DOI:** 10.1371/journal.pone.0247939

**Published:** 2021-04-08

**Authors:** Wiebe M. C. Top, Philippe Lehert, Casper G. Schalkwijk, Coen D. A. Stehouwer, Adriaan Kooy

**Affiliations:** 1 Department of Internal Medicine, Care Group Treant, Location Bethesda Hoogeveen, Hoogeveen, The Netherlands; 2 Bethesda Diabetes Research Center, Hoogeveen, The Netherlands; 3 Department of Statistics, Faculty of Economics, Facultés Universitaires Catholiques de Mons, Louvain Academy, Mons, Belgium; 4 Department of Internal Medicine and Cardiovascular Research Institute Maastricht, Maastricht University Medical Center, Maastricht, The Netherlands; 5 Department of Internal Medicine, University Medical Center Groningen, University of Groningen, Groningen, The Netherlands; Universidad Miguel Hernandez de Elche, SPAIN

## Abstract

**Background:**

Beyond antihyperglycemic effects, metformin may improve cardiovascular outcomes. Patients with type 2 diabetes often have an elevated plasma level of N-terminal pro B-type as a marker of (sub) clinical cardiovascular disease. We studied whether metformin was associated with a reduction in the serum level of N-terminal pro B-type natriuretic peptide (NT-proBNP) in these patients.

**Methods:**

In the HOME trial 390 insulin-treated patients with type 2 diabetes were randomized to 850 mg metformin or placebo three times daily. Plasma samples were drawn at baseline, 4, 17, 30, 43 and 52 months. In a post-hoc analysis we analyzed the change in NT-proBNP in both groups. We used a longitudinal mixed model analysis adjusting for age, sex and prior cardiovascular disease. In a secondary analysis we assessed a possible immediate treatment effect post baseline.

**Results:**

Metformin did not affect NT-proBNP levels over time in the primary analysis (-1% [95%CI -4;3, p = 0.62]). In the secondary analysis there was also no sustained time independent immediate treatment effect (initial increase of 17% [95%CI 4;30, p = 0.006] followed by yearly decrease of -4% [95%CI -7;0, p = 0.07]).

**Conclusions:**

Metformin as compared to placebo did not affect NT-proBNP plasma levels in this 4.3-year placebo-controlled trial. Potential cardioprotective effects of metformin cannot be explained by changes in cardiac pressures or volumes to the extent reflected by NT-proBNP.

## Background

Metformin is the established cornerstone of treatment in patients with early (around time of diagnosis) and advanced type 2 diabetes mellitus (T2D), with and without insulin therapy [1].

The HOME trial (Hyperinsulinaemia: the Outcomes of its Metabolic Effects), a 4.3 year placebo-controlled randomized trial in patients with advanced T2D, defined as failure of maximal oral antidiabetic treatment (HbA1c > 7.5%) and need for exogenous insulin, showed that metformin added to insulin prevented weight gain, improved glycemic control and reduced insulin requirements [2]. In addition, metformin improved endothelial function and decreased the occurrence of cardiovascular disease as an aggregated secondary endpoint [2, 3]. These findings are in line with the United Kingdom Prospective Diabetes Study (UKPDS), an open-label randomized controlled trial, which suggested favorable cardiovascular outcomes in obese patients with new-onset T2D treated with metformin [4].

The mechanisms by which metformin may improve cardiovascular outcomes are still unclear. In addition to an improvement in vascular endothelial function [3], metformin may have direct cardioprotective effects. Metformin has been shown to reduce acute myocardial reperfusion injury in animals and humans, and may improve diastolic function [5]. Both coronary artery disease and diastolic dysfunction are common in advanced T2D [6].

To further investigate potential cardioprotective effects of metformin, we hypothesized that metformin may reduce the blood levels of N-terminal pro B-type natriuretic peptide (NT-proBNP). NT-proBNP is a neurohormone secreted predominantly by cardiomyocytes in the left ventricle in response to volume expansion and pressure overload.

In patients with T2D, higher plasma levels of NT-proBNP have been found compared to those in the general population [7]. Apart from reduced renal clearance, this could be due to structural cardiac injury due to progressive diastolic failure and silent cardiac ischemia [8, 9].

In this study, we analyzed whether metformin versus placebo decreased NT-proBNP levels during a period of 4.3 years, in patients with advanced T2D on insulin therapy.

## Methods

This study is a post-hoc analysis of the HOME study, a randomized, placebo-controlled, double-blind multicenter trial. The HOME study is registered on clinicaltrials.gov with identifier NCT00375388. Adult T2D patients on insulin therapy with an estimated glomerular filtration rate (eGFR) > 50mL/min/1.73m2 were eligible as described previously [2]. Patients with congestive heart failure New York Heart Association class III/IV were excluded. Patients were recruited in three non-academic hospitals in the Netherlands. Patients meeting study criteria were, after informed consent 1:1 allocated to either metformin or placebo. The following medical ethical committees approved the trial protocol: the medical ethical committee of the Bethesda Hospital Hoogeveen, the medical ethical committee of the Deaconesses’ Hospital Meppel and the medical ethical committee of the Aleida Kramer Hospital Coevorden. After an initial washout period of 12 weeks in which oral antidiabetic drugs were stopped and patients were kept on insulin, 390 patients were randomized and followed for 4.3 years.

**Fig 1:**
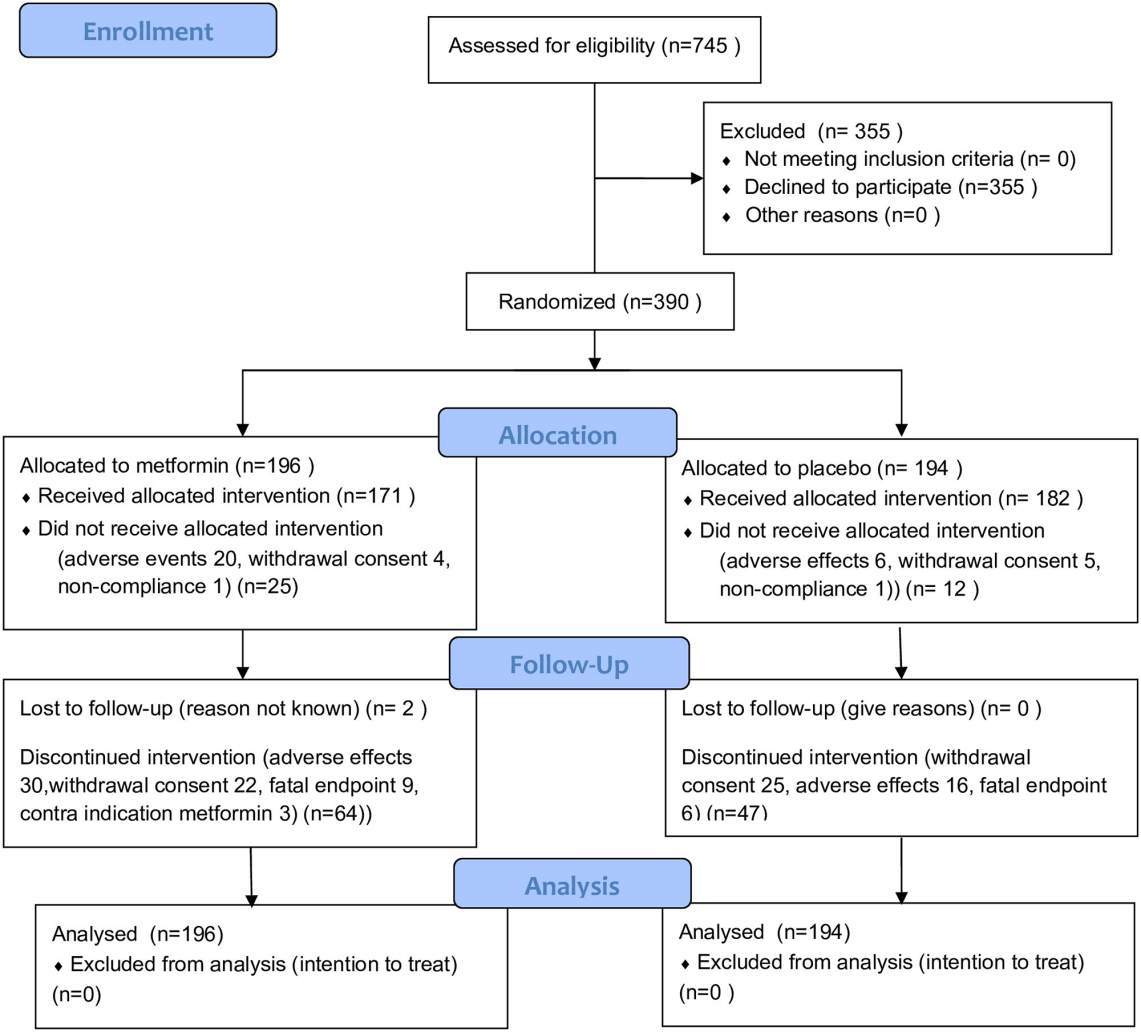
Flowchart.

The study was completed in 2002 with over 70% of the participants completing the planned follow-up. Major reasons for non-completing were adverse events (12%) and withdrawal of consent (8%) as shown in the flowchart (Fig 1). Mortality during the study was 4%.

Randomization was performed with a computer program, allocating random numbers to identical-looking boxes and tablets of either metformin or placebo.

Metformin 850 mg and placebo were titrated to a maximum tolerated dose of three times daily. In renal dysfunction the maximum dose was lowered according a predefined algorithm: in case of a Cockroft-Gault estimated glomerular filtration rate (eGFR) between 30–40 or 40–60 mL/min/1.73m^2^, the maximum dose was 850 mg once or twice daily, respectively.

NT-proBNP levels were available for 386 patients (99%) at baseline and for 256 patients (66%) at the final visit. The reason participants did not complete the full study period was mainly because of side-effects and withdrawal of informed consent. Plasma samples were stored at −80°C until analysis, with an immunoassay (MesoScaleDiscovery). The intra-assay variation was 4.9% and the inter-assay variation was 8.2%. Because NT-proBNP has a high biological variation and its distribution is positively skewed, we used the median as a summary statistic.

### Statistical analysis

We predefined the primary outcome as the change in NT-proBNP levels in metformin users as compared to placebo. Plasma samples were drawn at baseline, 4, 16, 28, 40 and 52 months.

All randomized patients were included in the analysis following the intention-to-treat principle.

The significance of metformin effect on NT-proBNP measurements was assessed by a longitudinal analysis featuring mixed model analysis of covariance. Due to the observed proportionality of means and standard deviations, NT-proBNP was log-normalized. Time and treatment (metformin versus placebo) were considered as fixed effects, patient as random factor, and age, sex and prior cardiovascular disease (the latter as a sum score) were used in the model as fixed covariates. Cardiovascular history is a sum score of cardiovascular events computed as myocardial infarction, cardiovascular intervention, amputation, stroke or transient ischemic attack, angina pectoris, dyspnea and claudication intermittens as earlier described [2]. Male sex at birth was coded as 1, female as 0. The significance of treatment was assessed by the first order interaction between time and treatment.

In secondary analyses, we also assessed the alternative hypothesis of a treatment effect from the first visit and remaining constant until the end of the trial [10] by assuming placebo effects at baseline and testing the treatment main effect. Also, we adjusted for eGFR and body mass index as additional covariates in the primary model.

All reported P values are 2-sided, and P < .05 was considered to be significant. The statistical analyses were performed using the software package R version 4.0.2 Package nlme (lme function) was used with default correlation settings (unstructured covariance).

## Results

**Fig 2:**
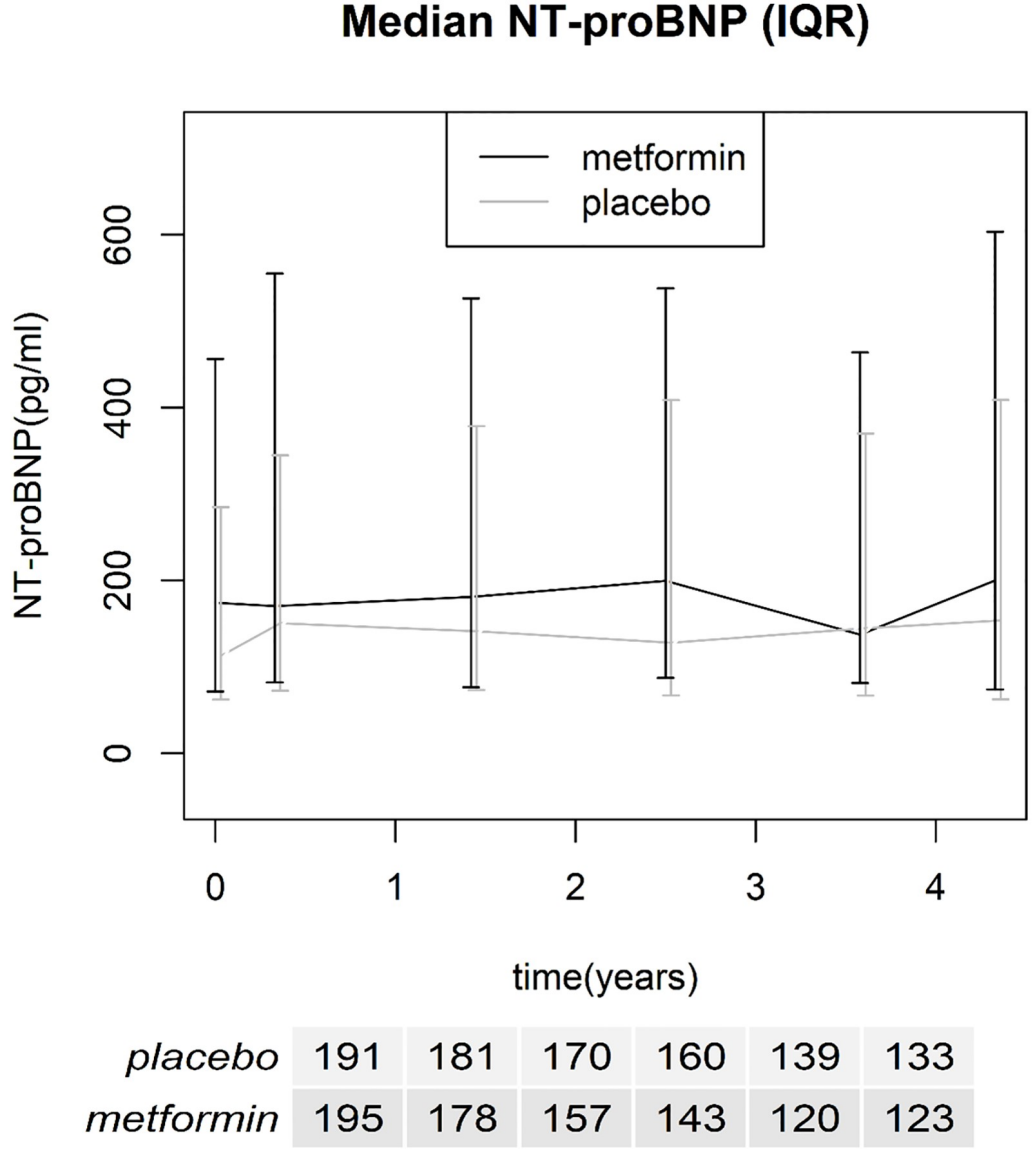
Median NT-proBNP over time with interquartile range, number of included patients for each time point is reported in the table below the x-axis.

A total of 196 subjects were randomized to receive metformin and 194 to receive placebo. The baseline demographic and clinical characteristics for each group are described in Table 1. Baseline median NT-proBNP was 140 pg/ml (IQR 64–347) for all patients. The median NT-proBNP levels for both treatment groups over time are shown in Fig 2, including number of patients included for each visit.

**Table 1:** Baseline characteristics.

	Placebo (n = 194)	Metformin (n = 196)
Demography		
Age, year	59.0(11.0)	63.5(9.61)
Men / women	97/97	81/115
Weight, kg	86.8 (15.0)	85.0 (15.6)
T2D duration, year	12.1 (7.93)	14.2 (8.67)
Diastolic BP, mm Hg	85.8 (11.1)	85.6 (12.1)
Systolic BP, mm Hg	159(24.6)	160(25.3)
Cardiovascular history	0.922 (1.33)	1.17 (1.43)
Daily dose of insulin, IU	63.9(25.5)	62.3 (28.7)
Lipid-lowering drugs (%)	31(16)	32(16)
BP-lowering drugs (%)	75(39)	93(47)
Plasma HbA1c, (%, [mmol/mol])	7.88 [63] (1.15)	7.87 [63] (1.21)
NT-proBNP, ng/l	113 [62.1,113]	174 [71.4,174]

Data are given as mean (SD) except NT-proBNP (median [interquartile range]), BP = blood pressure. Cardiovascular history is a sum score of cardiovascular events computed as myocardial infarction, cardiovascular intervention, amputation, stroke or transient ischemic attack, angina pectoris, dyspnea and claudication intermittens as earlier described [2].

Fixed effects of the model are shown in Table 2. NT-proBNP levels increased slowly over time. The time effect was an increase of 6% (95%CI 3;8, p<0.001) per year. NT-proBNP significantly and positively correlated with age (increase of 5% [95%CI 4;6, P<0.001] per year) and prior cardiovascular disease (increase of 29% [95%CI 19–39, P<0.001] per sum score point).

**Table 2:** Mixed model estimates.

	*Dependent variable*: log(NT proBNP)
Time effect	1.058 (1.033, 1.084) *
Treatment effect	0.972 (0.787, 1.201)
Treatment effect over time	0.991 (0.957, 1.027)
Age	1.050 (1.039, 1.060) *
Sex	1.102 (0.901, 1.346)
Cardiovascular severity score	1.287 (1.194, 1.389) *

Mixed model fixed effect estimates, data are back transformed as geometric mean ratios with corresponding 95% confidence intervals. Level of significance:

* p<0.001.

Metformin did not affect NT-proBNP levels over time in the primary analysis (-1% [95%CI -4;3, p = 0.62]). In the secondary analysis there was also no sustained time independent immediate treatment effect (initial increase of 17% [95%CI 4;30, p = 0.006] followed by yearly decrease of -4% [95%CI -7;0, p = 0.07]).

Addition of age and/or prior cardiovascular disease as interaction terms and adjustment for eGFR or body mass index did not change our results either. Also we respecified the correlation structure of our model with autoregressive correlation. The results remained the same (-1%[95%CI -5:3, p = 0.60).

In addition we did a per protocol analysis for the patients who completed their final visit. Results remained the same (-1% [95%CI -5:3, p = 0.66).

## Discussion

This is the first post-hoc analysis of a long-term placebo-controlled trial on the effects of metformin on NT-proBNP, a clinically relevant marker for CVD. In this study, we show that metformin does not significantly affect NT-proBNP in patients with advanced T2D.

Baseline NT-proBNP levels in our study were relatively high as compared to those in the general population and in a random T2D population [11]. Our population had advanced T2D with a significant burden of CVD. Such a high-risk population is suitable to detect a potential risk reduction by metformin, but also represents structural, cumulative and potentially irreversible myocardial injury translated in increased NT-proBNP levels, not responding to such a potentially risk-reducing treatment.

We confirm that age is positively correlated with NT-proBNP levels, independently of prior CVD. During the study, NT-proBNP levels increased steadily. The age dependency of NT-proBNP is well known, potentially due to subclinical cardiovascular disease increasing myocardial wall stress and affecting renal function and clearance of NT-proBNP [12].

In non-diabetic individuals with coronary heart disease, metformin (versus placebo) did not affect NT-proBNP during a period of 18 months [13]. Moreover, short-term treatment with metformin did not change NT-proBNP levels within 4 months and two years after a myocardial infarction [14].

Well-designed cardiovascular outcome trials with metformin in T2D patients are scarce. The UKPDS, a non-placebo-controlled, open label trial, was the first study showing a reduction of cardiovascular disease and cardiovascular mortality of 40% in obese T2D patients using metformin compared to controls [4]. We found a comparable reduction of cardiovascular disease in the placebo-controlled HOME study [2]. A third trial, the SPREAD DIMCAD trial showed a 46% reduction in recurrent cardiovascular events by metformin when compared to the active comparator glipizide [15].

Metformin might also be protective in diastolic heart failure. About 33% of T2D patients with preserved left-ventricle (LV) ejection fraction have signs of subclinical LV diastolic dysfunction. In the subgroup of suboptimal glycemic control the incidence increases to 40% [6]. Animal studies show that metformin lowers passive stiffness of the heart muscle in a mouse model, improving diastolic function. This was confirmed in humans by Andersson showing an improved LV relaxation in patients on metformin in a retrospective study [5].

The absence of an effect of metformin on NT-proBNP does not imply the absence of an effect of metformin on cardiovascular risk. The PONTIAC trial showed that cardiovascular risk can change without a corresponding change in the NT-proBNP [16]. NT-proBNP can be used to guide and follow therapy but this holds primarily true for heart failure with reduced ejection fraction.

Limitations of the present study are the lack of other cardiovascular biomarkers than NT-proBNP and the absence of (echocardiographic) measures of cardiac function.

Strengths of the present study are the placebo-controlled study design, the long period of follow-up of 52 months (4.3 years), the large numbers of samples supporting the robustness of the results and the design of the trial without antihyperglycemic agents other than metformin and insulin.

## Conclusions

Metformin did not affect NT-proBNP levels during the 4.3 year follow up despite the earlier described reduction of cardiovascular disease in the HOME trial. Increases of NT-proBNP levels due to cumulative cardiovascular disease and aging might have become irreversible over time. The potential cardioprotective effects of metformin cannot be explained by reversible changes in myocardial pressures or volumes to the extent reflected by NT-proBNP.

## Supporting information

S1 Checklist (DOC)

S1 File Interim analysis Wulffele 2002. (PDF)

S2 File Original trial Kooy 2009. (PDF)

S3 File Study protocol HOME. (PDF)
