# Supplementary material for: Metformin and N-terminal pro B-type natriuretic peptide in type 2 diabetes patients, a post-hoc analysis of a randomized controlled trial
Source: PLoS One. 2021 Apr 8;16(4):e0247939. doi: 10.1371/journal.pone.0247939 (PMC8031400; doi:10.1371/journal.pone.0247939)
Supplement: S1 File — (PDF) [file pone.0247939.s002.pdf]

Combination of Insulin and Metformin in the Treatment of Type 2 Diabetes

MICHIEL G. WULFFELÉ, MD[1]
ADRIAAN KOOY, MD, PHD[1]
PHILIPPE LEHERT, PHD[2]
DANIEL BETS, MSC[3]
JELES C. OGTEROP, MD[4]
BOB BORGER VAN DER BURG, MD[5]
AB J.M. DONKER, MD, PHD[6]
COEN D.A. STEHOUWER, MD, PHD[6]

OBJECTIVE — To investigate the metabolic effects of metformin, as compared with placebo, in type 2 diabetic patients intensively treated with insulin.

RESEARCH DESIGN AND METHODS — Metformin improves glycemic control in poorly controlled type 2 diabetic patients. Its effect in type 2 diabetic patients who are intensively treated with insulin has not been studied. A total of 390 patients whose type 2 diabetes was controlled with insulin therapy completed a randomized controlled double-blind trial with a planned interim analysis after 16 weeks of treatment.The subjects were selected from three outpatient clinics in regional hospitals and were randomly assigned to either the placebo or metformin group, in addition to insulin therapy. Intensive glucose monitoring with immediate insulin adjustments according to strict guidelines was conducted. Indexes of glycemic control, insulin requirements, body weight, blood pressure, plasma lipids, hypoglycemic events, and other adverse events were measured.

RESULTS — Of the 390 subjects, 37 dropped out (12 in the placebo and 25 in the metformin group). Of those who completed 16 weeks of treatment, metformin use, as compared with placebo, was associated with improved glycemic control (mean daily glucose at 16 weeks 7.8 vs. 8.8 mmol/l, $P = 0.006$; mean GHb 6.9 vs. 7.6%, $P < 0.0001$); reduced insulin requirements (63.8 vs. 71.3 IU, $P < 0.0001$); reduced weight gain (−0.4 vs. +1.2 kg, $P < 0.01$); and decreased plasma LDL cholesterol (−0.21 vs. −0.02 mmol/l, $P < 0.01$). Risk of hypoglycemia was similar in both groups.

CONCLUSIONS — In type 2 diabetic patients who are intensively treated with insulin, the combination of insulin and metformin results in superior glycemic control compared with insulin therapy alone, while insulin requirements and weight gain are less.

Diabetes Care 25:2133–2140, 2002

The U.K. Prospective Diabetes Study (UKPDS) (1–3) has shown that tight glycemic control in type 2 diabetes substantially decreases the risk of diabetes-related complications. However, the best way to achieve tight glycemic control is not clear. Among the oral antihyperglycemic agents, metformin may offer certain advantages. First, the use of metformin is associated with less weight gain (2). Second, metformin has several other effects that may decrease the risk of atherothrombotic disease—it is associated with reduced insulin requirements, and reduced blood levels of insulin (4), LDL cholesterol (5), PAI-1 (6), and methylglyoxal (7), which is a precursor of advanced glycation end products. Finally, metformin may also decrease blood pressure (4,8). However, metformin use is also associated with an increase in plasma homocysteine concentration (9,10), an increased mortality risk when combined with a sulfonylurea derivative (2), and an increased risk of lactic acidosis although the absolute risk is low (11).

A significant number of patients with type 2 diabetes cannot achieve tight glycemic control with oral agents and need to be treated with insulin, either as a single agent or added to an oral regimen. The combination of insulin with sulfonylurea derivatives has been extensively studied (12), but there are few data on combination therapy with metformin and insulin. Four small placebo-controlled trials, conducted in the outpatient clinics of university medical centers (4,13–15), reported that the addition of metformin to insulin in patients with poorly controlled type 2 diabetes resulted in improved glycemic control and reduced insulin requirements, while preventing weight gain.

The randomized, placebo-controlled trial Hyperinsulinemia: the Outcome of its Metabolic Effects (HOME) was designed to investigate, in a nonacademic setting, whether metformin decreases cardiovascular morbidity in patients with type 2 diabetes treated with insulin to achieve tight glycemic control (defined as a GHb <7%) (16), during a follow-up of 4.3 years. An interim analysis was done after 16 weeks of treatment to see whether the goal of tight glycemic control was met. In this article we report unexpected favorable effects of metformin on glycemic control during an intensive regimen of insulin therapy.

RESEARCH DESIGN AND METHODS

Patients and procedures

We aimed to include 400 patients with type 2 diabetes between 30 and 80 years

From the [1]Department of Internal Medicine, Bethesda Hospital Hoogeveen, Drenthe, the Netherlands; the [2]Department of Biostatistics, University of Mons, Mons, Belgium; the [3]Clinical Research and Development, E. Merck Nederland B.V. Amsterdam, Noord Holland, the Netherlands; the [4]Department of Internal Medicine, Deaconesses' Hospital Meppel, Drenthe, the Netherlands; the [5]Department of Internal Medicine, Aleida Kramer Hospital Coevorden, Drenthe, the Netherlands; and the [6]Department of Internal Medicine and Institute for Cardiovascular Research, Vrije Universiteit Medical Centre, Amsterdam, Noord Holland, the Netherlands.

Address correspondence and reprint requests to Adriaan Kooy, MD, PhD, Bethesda Hospital, 7909 AA Hoogeveen, The Netherlands. E-mail: kooy.a@bethesda.nl.

Received for publication 24 October 2001 and accepted in revised form 9 September 2002.

P.L. is an occasional consulting statistician for Merck.

Abbreviations: HOME, Hyperinsulinemia: the Outcome of its Metabolic Effects; UKPDS, U.K. Prospective Diabetes Study.

A table elsewhere in this issue shows conventional and Système International (SI) units and conversion factors for many substances.

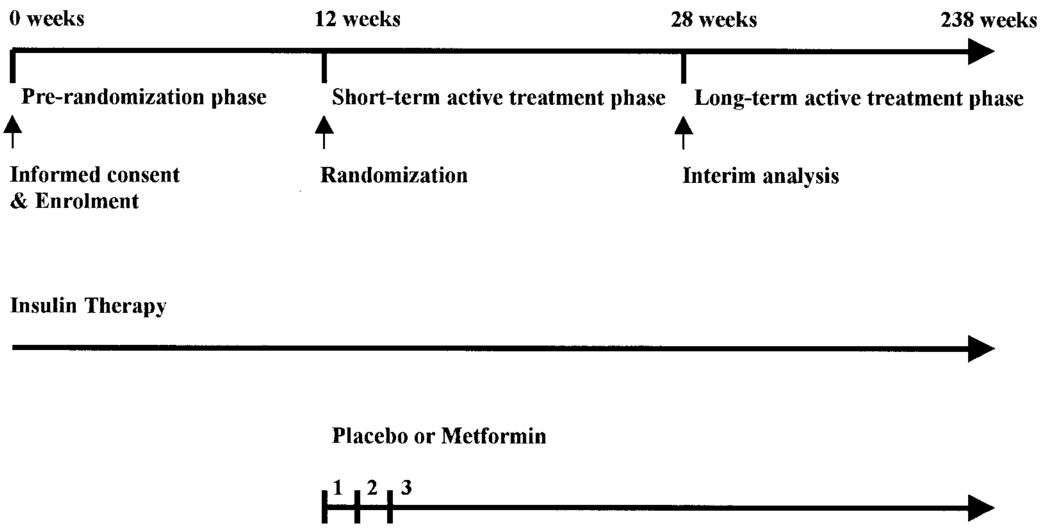

Procedures and goals

Prerandomization phase

- Optimization of glycemic control
- Cessation of antihypertensive and lipid lowering medication
- Cessation of metformin treatment (n=45)

Short-term active treatment phase

- Determination of baseline characteristics, including a complete medical history and physical examination at week 12
- Less tight treatment of blood pressure and plasma LDL cholesterol
- Randomization to metformin or placebo
- Collection of data on outcome variables plus a repeat complete medical history and physical examination at week 28

Long-term active treatment phase

- Optimization of glycemic control
- Tight treatment of blood pressure and plasma LDL cholesterol
- Repeat complete medical history and physical examination at 3-month intervals

Figure 1—*HOME-trial schedule.*

of age who had received a diagnosis of diabetes after 25 years of age, had never had an episode of ketoacidosis, and whose blood glucose–lowering treatment had previously consisted of oral agents but now exclusively consisted of insulin or a combination of insulin and metformin. We excluded pregnant women and women trying to become pregnant, patients with a Cockroft-Gault–estimated creatinine clearance <50 ml/min (17) or low plasma cholinesterase (reference value, ≥3.5 units/l) (18), and patients with congestive heart failure (New York Heart Association class III/IV) or other serious medical or psychiatric diseases.

All patients gave written informed consent. The medical ethical committees of the three participating hospitals approved the trial protocol. The trial has been and is conducted in accordance with the Note for Guidance on Good Clinical Practice (CPMP/ICH/135/95) dated 17 July 1996 and in accordance with the Declaration of Helsinki (revised version of Hong Kong, 1989).

Study design

The HOME trial was conducted in the outpatient clinics of three nonacademic hospitals (Hoogeveen, Meppel, and Coevorden). The trial design consisted of three phases (Fig. 1). In the 12-week prerandomization phase we aimed to optimize glycemic control by intensive glucose monitoring and insulin adjustment (target plasma glucose levels between 4 and 7 mmol/l in the fasting state and between 4 and 10 mmol/l postprandially). All subjects monitored their plasma glucose levels at home every 2 weeks (i.e., just before and ~90 min after breakfast, lunch, and dinner and at bedtime) using the same monitoring device (Glucotouch; Lifescan, Beerse, Belgium). The patients reported these values by telephone to a nurse specialized in diabetes care, who, if necessary, gave advice to adjust the insulin dose or try another insulin mixture or injection schedule. All patients were treated with insulin four times daily (Actrapid preceding the three meals and Insulatard ante noctem; Novo Nordisk, Alphen a/d Ryn, the Netherlands) or twice daily (Mixtures of Actrapid (10–50%) and Insulatard (90–50%) preceding breakfast and dinner: Mixtard preparations; Novo Nordisk). Individual titration took place according to good clinical practice to reach the target glucose levels and to prevent hypoglycemia. The nurse specialized in diabetes care, and if necessary, gave advice to adjust the insulin dose or

Table 1—*Baseline characteristics*

	Placebo	Metformin
Demographics		
n	182	171
Men/women	91/91	76/95
Age (years)	58.9 ± 11.1	63.2 ± 9.8
Currently smoking	54 (30)	36 (21)
Duration of diabetes (years)	12.0 ± 8.0	14.0 ± 8.4
Insulin treatment (years)	5.7 ± 5.9	6.5 ± 7.5
Diabetic complications		
Cardiovascular	53 (29)	59 (35)
Retinal coagulation and (or) cataract extraction	25 (14)	38 (22)
Amputation	3 (2)	4 (2)
Paresthesias	79 (43)	83 (49)
Concomitant medication		
Lipid-lowering drugs	36 (20)	34 (20)
Blood pressure–lowering drugs	73 (40)	88 (51)
Clinical features		
Waist-to-hip ratio		
Men	1.03 ± 0.09	1.02 ± 0.08
Women	0.92 ± 0.09	0.93 ± 0.1

Data are mean ± SD or *n* (%).

try another insulin mixture (e.g., Mixtard 50/50 instead of Mixtard 30/70) or injection schedule (e.g., four times instead of twice daily). The adjustments of the insulin dose took place in "small steps," changing the dose by ≤4 units per injection. If the target values for glycemic control were difficult to reach, the study nurse consulted the principal investigator for advice to optimize the insulin therapy. This intensive glucose-monitoring and insulin adjustment scheme was continued during the whole trial. The second feature of the prerandomization phase was the discontinuation of concomitant medication for hypertension and dyslipidemia if the patient's physician approved this.

At the start of the 16-week short-term active treatment phase, all subjects were randomly assigned in a double-blind fashion to receive placebo or metformin in addition to insulin therapy. All patients were numbered in order of study entry and received trial medication with the same number. The boxes and tablets of metformin and placebo had a similar appearance. Each subject successively increased the dose from one to finally three tablets a day, if tolerated. The first tablet was taken at bedtime, the second at breakfast, and the third at dinner. The dose of the metformin tablets was 850 mg, and the actual mean dose in the metformin-treated group was 2,163 mg during the trial. At the beginning and at the end of the short-term active treatment phase, fasting blood samples were drawn, a physical examination was carried out, and a complete medical history was taken. During the prerandomization and short-term active treatment phases, target values for blood pressure, urinary albumin-to-creatinine ratio, and plasma LDL cholesterol concentration were <180/110 mmHg, <100 mg/mmol, and <7.0 mmol/l, respectively, because we wished to study the effects of metformin on these variables in the (near) absence of blood pressure– and cholesterol-lowering treatment. The less-tight blood pressure and cholesterol targets that were thought acceptable according to Dutch guidelines during the development of the study protocol and during the first year of the trial (1996–1997) would not be considered acceptable today (19,20).

The 48-month long-term active treatment phase is a continuation of the short-term active treatment phase. However, in this phase, we aimed for tight control of blood pressure, urinary albumin-to-creatinine ratio, and plasma LDL cholesterol levels (target values <150/90 mmHg, <3.5 mg/mmol in women and <2.5 mg/mmol in men, and <2.6 mmol/l, respectively).

The specified medications used for the treatment of hyperlipidemia were simvastatin and acipimox. The medications used for the treatment of hypertension were enalapril, kaliumlosartan, hydrochlorthiazide, and lercanidipine. Both groups were comparably treated with these drugs according to a specified "add-on protocol" to reach the target values. No significant differences were found between the treatment groups in the use of these concomitant medications. The results presented here exclusively concern the prerandomization and short-term active treatment phases.

Physical examination

The same investigator carried out all examinations. We measured blood pressure, weight, height, and waist and hip circumferences and calculated the BMI (kg/m^2) and the waist-to-hip ratio (dimensionless). Patients were weighed wearing underclothes. The initial blood pressure measurement was taken at both arms after the patient had sat down for at least 10 min. Follow-up measurements were taken at the side where the highest systolic pressure was noted. We used a mercury sphygmomanometer (Speidel-Keller, miniatur 300) with several cuffs, depending on the size of the subject's arm.

Laboratory investigations

The laboratories of the three hospitals used standard analytical methods with the same reference values for all laboratory variables. Plasma glucose levels were determined using an automated glucose oxidase method (Hitachi 917; Roche, Basel) in Hoogeveen and Meppel. GHb (normal value 4.0–6.0%) was measured by high-performance liquid chromatography in Hoogeveen and by an immunoturbidimetric method (Unimate; Roche) in Meppel. Method comparison according to Passing and Bablok (21,22) showed no significant deviation between these methods. Moreover, by using a randomized Block test on GHb values no significant difference could be found between the laboratories. Fasting lipid and lipoprotein concentrations were assessed by standard methods. Plasma LDL cholesterol was calculated with use of the Friedewald formula if triglycerides were <4.5 mmol/l (23). The Coevorden Hospital used dry chemistry for all the above-mentioned laboratory measurements

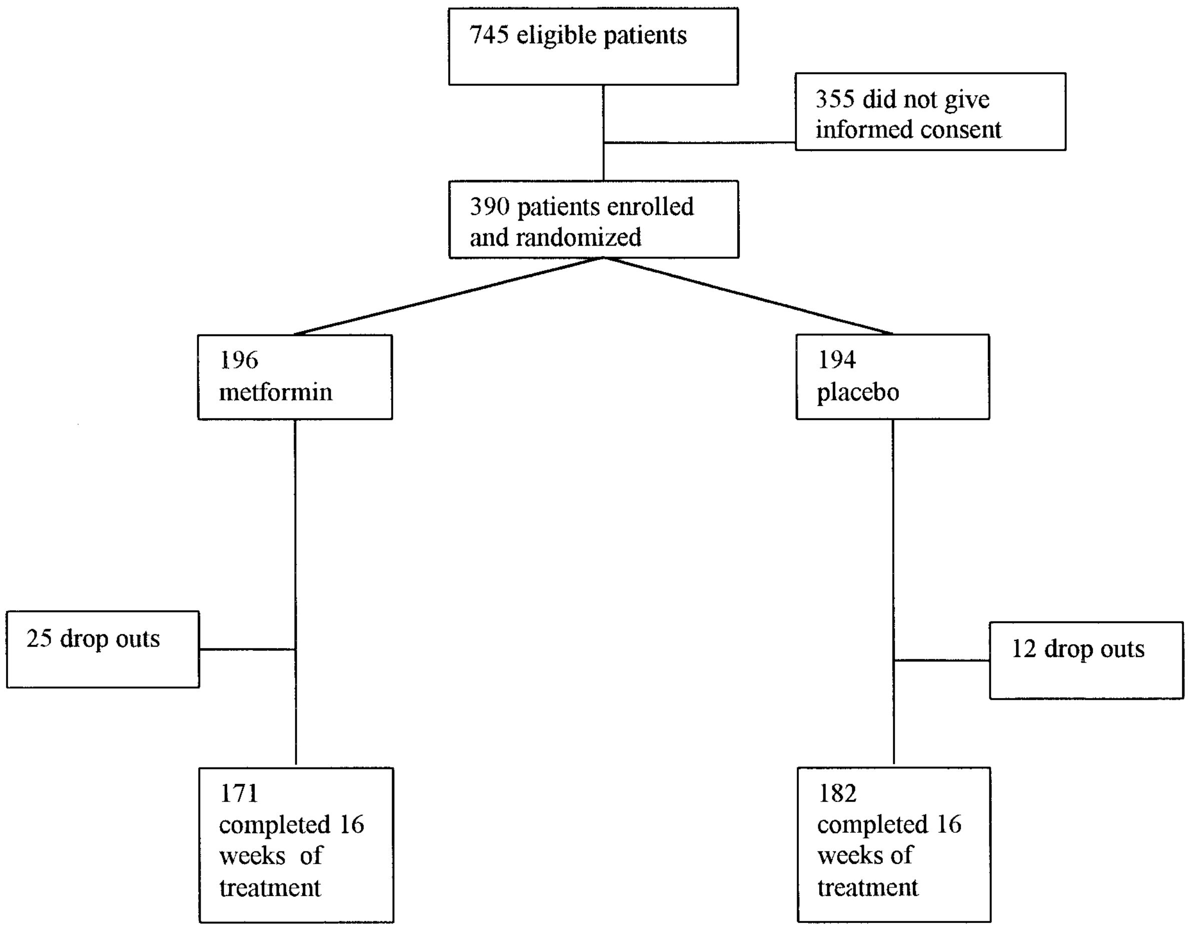

Figure 2—*Trial profile.*

(Orthoclinical Diagnostics; Johnson and Johnson, Rochester, NY).

Statistical analysis

A power analysis indicated that 390 patients needed to be randomized to demonstrate a difference in the incidence of micro- and macrovascular events of 8% points between the groups after 4 years of follow-up (one-tailed test with an α of 0.05 and a β of 0.25; expected cumulative incidences of cardiovascular events 20 vs. 12% in the placebo and metformin groups, respectively). However, in this interim analysis the primary end points are GHb and the daily dose of insulin. Secondary end points are BMI, body weight, plasma cholesterol, plasma triglycerides, and blood pressure.

The data presented concern all patients seen at the beginning and the end of the short-term active treatment phase. In addition, we did an intent-to-treat analysis in which we assumed that outcome variables in patients who dropped out did not change between baseline and follow-up. As the HOME-trial is ongoing, the treatment codes were not disclosed to the investigators and were available only to the statistician, who provided only grouped data with means and SD to the investigators. According to the planned protocol, the significance of the effects of metformin, as compared with placebo, on the quantitative end points (plasma glucose levels, GHb, insulin requirement, weight, blood pressure, and plasma lipids) was assessed by analyzing the final value, adjusting for the baseline value through an ANCOVA (24). The χ^2 test was used to compare the number of hypoglycemic events between the two treatment groups. Data are expressed as mean (SD) or mean (95% CI). The median and 25th and 75th percentiles are given for insulin dosage. All tests were two-sided and differences with $P < 0.05$ were considered to be statistically significant. A supplementary multiple regression analysis was performed with age as covariate and its first order interaction with all outcome variables. Double data entry and statistical analyses were carried out with the SAS package 6.08 on the Windows system.

RESULTS

Patients

We screened the medical files of all three participating outpatient clinics and identified 745 eligible patients. All were approached to enroll into the trial and 390 subjects gave written informed consent (Fig. 2). The subjects were randomized to receive metformin (196 subjects) and placebo (194 subjects). Of the 390 subjects, 37 dropped out, 25 in the metformin and 12 in the placebo group. A total of 2 patients never took their medication (placebo 1, metformin 1), 9 withdrew their consent (placebo 5, metformin 4), and 26 experienced adverse effects (placebo 6, metformin 20). Of these 26, 11 experienced diarrhea (placebo 2, metformin 9),

Table 2—*Changes in outcome parameters: blood pressure, plasma lipids, GHb, body weight, and BMI*

	Baseline		Follow-up		Change		
Outcome parameter	Placebo	Metformin	Placebo	Metformin	Placebo	Metformin	*P*
Systolic blood pressure (mmHg)	159 ± 25	160 ± 26	161 ± 25	161 ± 25	2.1 ± 17	1.8 ± 17	0.87
Diastolic blood pressure (mmHg)	86 ± 11	85 ± 12	88 ± 14	88 ± 13	2.6 ± 11	2.5 ± 11	0.92
Total cholesterol (mmol/l)	5.49 ± 1.23	5.58 ± 1.13	5.45 ± 1.23	5.31 ± 0.99	−0.04 ± 0.75	−0.25 ± 0.65	0.006
HDL cholesterol (mmol/l)	1.26 ± 0.41	1.31 ± 0.41	1.26 ± 0.40	1.30 ± 0.39	0 ± 0.17	−0.01 ± 0.18	0.79
LDL cholesterol (mmol/l)	3.40 ± 1.02	3.54 ± 1.03	3.37 ± 1.14	3.27 ± 0.98	−0.05 ± 0.72	−0.24 ± 0.63	0.01
Triglycerides (mmol/l)	1.88 ± 1.53	1.66 ± 1.13	1.90 ± 1.53	1.63 ± 1.13	0.02 ± 1.21	−0.02 ± 0.87	0.75
GHb (% Hb)	7.88 ± 1.21	7.86 ± 1.17	7.61 ± 1.17	6.94 ± 0.98	−0.27 ± 0.84	−0.91 ± 0.93	<0.0001
Daily dose of insulin (IU/day)	69.9 ± 33.2	71 ± 33.1	71.3 ± 33.2	63.8 ± 40.3	1.4 ± 9	−7.2 ± 10	<0.0001
Body weight (kg)	86.2 ± 14.6	85.6 ± 15.7	87.4 ± 16.1	85.1 ± 16	1.2 ± 5.3	−0.4 ± 2.5	<0.0001
BMI (kg/m^2)	29.5 ± 4.6	29.9 ± 5.2	30 ± 5.4	29.7 ± 5.3	0.4 ± 2	−0.2 ± 0.9	0.001

Data are mean ± SD.

5 flatulence (placebo 1, metformin 4), 4 fatigue (placebo 1, metformin 3), 1 pruritus (metformin), 1 headaches (metformin), 1 pyrosis (placebo), 1 nausea (metformin), 1 myocardial infarction (placebo), and 1 patient died suddenly (metformin).

Table 1 shows baseline characteristics at the start of the short active treatment phase. Patients randomized to metformin were slightly older than patients randomized to placebo (63.2 ± 9.8 vs. 58.9 ± 11.1 years), but other baseline characteristics were comparable between the two groups.

Glycemic control and daily insulin requirement

In the short-term active treatment phase, plasma glucose values obtained with home monitoring decreased significantly more in the metformin group than in the placebo group (Fig. 3). The mean daily glucose level decreased from 8.8 ± 2.1 to 8.5 ± 1.7 mmol/l in the placebo group (mean decrease −0.16; 95% CI −0.53 to 0.22 mmol/l) and from 8.8 ± 2.2 to 7.8 ± 1.7 mmol/l in the metformin group (P = 0.006 vs. placebo; mean decrease −1.04; 95% CI −1.5 to −0.52 mmol/l). The mean daily glucose level decreased 0.7 mmol/l more in the metformin group than in the placebo group.

Changes in GHb and daily dose of insulin are shown in Table 2. Mean GHb level decreased from 7.9 ± 1.2 to 7.6 ± 1.2% in the placebo group (P = 0.031 vs. baseline; mean decrease −0.27; 95% CI −0.25 to −0.52% point) and from 7.9 ± 1.2 to 6.9 ± 1.0% in the metformin group (P < 0.0001 vs. baseline vs. placebo; mean decrease −0.91; 95% CI −0.69 to −1.15% point). Mean GHb levels decreased 0.6% point more in the metformin than in the placebo group (Table 2). The difference in GHb levels between the metformin and placebo groups was similar in each of the three centers (P for interaction = 0.51, data not shown). The daily dose of insulin increased 1.4 IU (95% CI 0.3–2.9) in the placebo group and decreased 7.2 IU (95% CI −5.8 to −8.8) in the metformin group (P < 0.0001 vs. placebo).

Body weight and BMI

Changes are shown in Table 2. In the placebo group, body weight and BMI increased by +1.2 kg (95% CI 0.4–2.0) and +0.4 kg/m^2 (95% CI 0.2–0.7), respectively. In the metformin group, body weight and BMI decreased by −0.4 kg (95% CI −0.1 to −0.8) and −0.2 kg/m^2 (95% CI −0.02 to −0.3), respectively (P < 0.01 vs. placebo for both; Table 2). Compared with the placebo group, the change in body weight was −1.6 kg and the change in BMI was −0.6 kg/m^2 in the metformin group.

Blood pressure and plasma lipids

Data are shown in Table 2. There was a small but nonsignificant increase in blood pressure in both groups that did not differ between the groups (Table 2). In the placebo group, plasma total and LDL cholesterol concentrations decreased by −0.04 mmol/l (95% CI −0.15 to 0.07) and −0.02 mmol/l (95% CI −0.16 to 0.06), respectively. In the metformin group, plasma total cholesterol and LDL cholesterol concentrations decreased by −0.25 (95% CI −0.35 to −0.15) and −0.21 mmol/l (95% CI −0.33 to −0.15), respectively (P < 0.01 vs. placebo for both). The differences between the metformin and the placebo group were −0.21 mmol/l for total cholesterol and −0.19 mmol/l for LDL cholesterol. LDL cholesterol concentrations were not calculated in patients with triglyceride values >4.5 mmol/l at baseline and/or at follow-up (n = 23 in the placebo and n = 18 in the metformin group). Adjustment for GHb change did not importantly change on the effect estimate of metformin treatment on plasma LDL cholesterol (data not shown). There were no significant changes in plasma HDL cholesterol and triglyceride concentrations in either group (data not shown).

Additional analyses

Secondary analyses were carried out. First, we reanalyzed the same results following the same ANCOVA model but using age as an additional covariate (because age was not totally balanced among groups at baseline). Second, to make sure that the results are independent of the initial intent-to-treat selection, we carried out the same analysis on the full set of patients, where for patients lost after baseline, final values were considered as unchanged with respect to baseline values. For these two supplemental analyses, the results, CIs, and corresponding P values were unchanged.

Adverse events and hypoglycemic incidents

In the short-term active treatment phase, the number of symptomatic hypoglycemic events per patient per month increased from 0.29 ± 0.83 to 1.12 ± 2.67

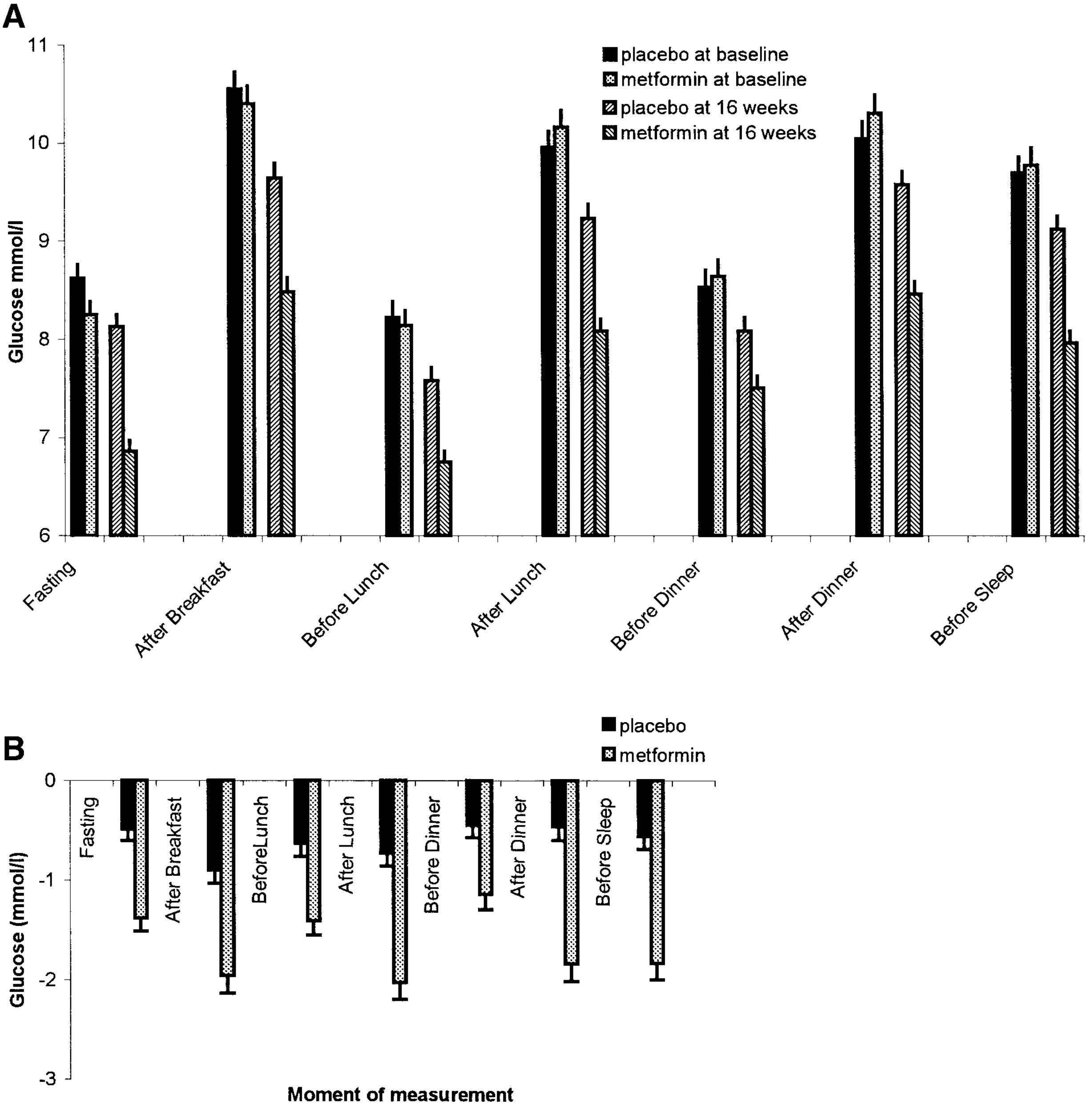

Figure 3—A*: Blood glucose levels measured at home.* B*: Change in blood glucose levels measured at home. Data are means with SD error bars. For each time point indicated, the first and the second bars show values at baseline and the third and the fourth show values at 16 weeks. Blood glucose levels in the metformin group compared with the placebo group are all significantly lower at 16 weeks* ($P < 0.05$)*. The change in glucose values is also significantly greater in the metformin than in the placebo group at all times during the day* ($P < 0.01$).

($P < 0.0001$) in the placebo group and from 0.86 ± 1.76 to 1.52 ± 4.29 (P = 0.06) in the metformin group (P = 0.29 vs. placebo). The occurrence of none to four or more hypoglycemic events per subject per month was comparable between the groups (none: placebo 123 [68%] vs. metformin 108 [64%]; one: placebo 30 [16%] vs. metformin 31 [18%]; two: placebo 11 [6%] vs. metformin 12 [7%]; three: placebo 11 [6%] vs. metformin 9 [5%]; four or more: placebo 7 [4%] vs. metformin 11 [6%]; χ^2 P = 0.477). Eight events in the metformin group and four in the control group required partner assistance, and none required medical assistance. Most patients tolerated the trial medication well (metformin 90%; placebo 97%). Mild and transient gastrointestinal complaints were reported most frequently in the metformin group (56%) and less in the placebo group (13%, $P < 0.0001$ vs. metformin).

CONCLUSIONS— We designed this placebo-controlled randomized double-blind trial to test the hypothesis that in individuals with type 2 diabetes who are intensively treated with insulin and reach similar glycemic control, the use of metformin to decrease the required daily dose of exogenous insulin is associated with a

decreased risk of cardiovascular disease. A planned interim analysis after 16 weeks of treatment showed that despite intensive glucose monitoring and frequent adjustment of the insulin dose, metformin use compared with placebo was associated with a statistically significant and potentially clinically relevant improvement of glycemic control (GHb 6.9 vs. 7.6%). Metformin use was also associated with less weight gain (−1.6 kg compared with placebo) and with a small decrease in LDL cholesterol (−0.19 mmol/l). Taken together, these data suggest that metformin may have important advantages in type 2 diabetic patients who are intensively treated with insulin. Our results were obtained in a nonacademic setting and may thus have broad applicability. Others recently suggested advantageous effects of metformin in poorly controlled type 2 diabetic patients treated in academic centers (4,13,14).

It is not clear why we did not achieve our aim of similar glycemic control in both groups. The groups were comparable at baseline, and compliance with the intensive treatment regimen was high. One possibility is that an increased risk of hypoglycemic events in the placebo (insulin only) group prevented further increments in the insulin dose. However, the occurrence of hypoglycemic events did not differ between the metformin and placebo groups (Table 2), and advice to increase the insulin dose was never refused in either group (data not shown). These data suggest that in type 2 diabetic patients treated with insulin, metformin may affect glucose metabolism in a way that is not easily achieved with exogenous insulin, for example by more efficiently reducing hepatic glucose output (25,26). This hypothesis requires further investigation.

Metformin use, compared with placebo, was associated with a 10% decrease of the daily dose of insulin, while glycemic control was better. This reduction in insulin requirement is consistent with that reported in earlier studies of obese type 2 diabetic patients with poor glycemic control (decrease varying from 4 to 25%) (4,13,27). A much longer follow-up is necessary to investigate whether this reduction in exogenous insulin requirement will result in a decreased risk of atherothrombotic disease (28–30). However, if less weight gain will occur, and be maintained during the longer term, in patients treated with less exogenous insulin, it will be difficult to unravel the contributions of weight and insulin dose on the incidence of cardiovascular disease. Therefore, we realize that favorable effects on cardiovascular disease (if any) must then be ascribed to the totality of effects of metformin and cannot be solely ascribed to the decrease in insulin dose. No definitive evidence yet exists that hyperinsulinemia itself is a significant atherotrombotic risk factor.

Our findings confirm a small but significant decrease of plasma LDL cholesterol during metformin treatment (30), which could not be explained by improvement in glycemia (5,31–34). Indeed, some in vitro studies show a direct effect of metformin on cholesterol metabolism (35,36).

In conclusion, our findings show that in type 2 diabetic patients who are intensively treated with insulin, the use of metformin is associated with improved glycemic control, a reduced insulin requirement, less weight gain, and a small decrease in LDL cholesterol levels. Metformin therefore is a valuable treatment option in insulin-treated type 2 diabetic patients.

Acknowledgments— This part of the HOME-trial was supported by grants from Byk, Lifescan, E. Merck Lipha, Merck Sharpe & Dohme, and Novo Nordisk.

We thank the study nurses Liesbeth Breedland and Els van Driesum for their dedication to the concerns of the patients and the quality of the treatments; Jan van der Kolk for his technical assistance and validation of the laboratory techniques; Gerard de Groot, Rob Hoorn, and Erk Pieterse for their hospitality at their laboratories; and all other members of the HOME-study group for their contribution to the HOME-trial.

References

1. U.K. Prospective Diabetes Study (UKPDS) Group: Intensive blood-glucose control with sulphonylureas or insulin compared with conventional treatment and risk of complications in patients with type 2 diabetes (UKPDS 33). *Lancet* 352:837–853, 1998
2. U.K. Prospective Diabetes Study (UKPDS) Group: Effect of intensive blood-glucose control with metformin on complications in overweight patients with type 2 diabetes (UKPDS 34). *Lancet* 352:854–865, 1998
3. Stratton IM, Adler AI, Neil HA, Matthews DR, Manley SE, Cull CA, Hadden D, Turner RC, Holman RR: Association of glycaemia with macrovascular and microvascular complications of type 2 diabetes (UKPDS 35): prospective observational study. *BMJ* 321:405–412, 2000
4. Giugliano D, Quatraro A, Consoli G, Minei A, Ceriello A, De Rosa N, D'Onofrio F: Metformin for obese, insulin-treated diabetic patients: improvement in glycaemic control and reduction of metabolic risk factors. *Eur J Clin Pharmacol* 44:107–112, 1993
5. DeFronzo RA, Goodman AM: Efficacy of metformin in patients with non-insulin-dependent diabetes mellitus: the Multicenter Metformin Study Group. *N Engl J Med* 333:541–549, 1995
6. Nagi DK, Yudkin JS: Effects of metformin on insulin resistance, risk factors for cardiovascular disease, and plasminogen activator inhibitor in NIDDM subjects: a study of two ethnic groups. *Diabetes Care* 16:621–629, 1993
7. Beisswenger PJ, Howell SK, Touchette AD, Lal S, Szwergold BS: Metformin reduces systemic methylglyoxal levels in type 2 diabetes. *Diabetes* 48:198–202, 1999
8. Landin K, Tengborn L, Smith U: Treating insulin resistance in hypertension with metformin reduces both blood pressure and metabolic risk factors. *J Intern Med* 229:181–187, 1991
9. Aarsand AK, Carlsen SM: Folate administration reduces circulating homocysteine levels in NIDDM patients on long-term metformin treatment. *J Intern Med* 244: 169–174, 1998
10. Carlsen SM, Folling I, Grill V, Bjerve KS, Schneede J, Refsum H: Metformin increases total serum homocysteine levels in non-diabetic male patients with coronary heart disease. *Scand J Clin Lab Invest* 57: 521–527, 1997
11. Wilholm B, Myrhed M: Metformin-associated lactic acidosis in Sweden 1977–1991. *Eut J Clin Pharmacol* 44:589–591, 1993
12. Johnson JL, Wolf SL, Kabadi UM: Efficacy of insulin and sulfonylurea combination therapy in type II diabetes: a meta-analysis of the randomized placebo-controlled trials. *Arch Intern Med* 156:259–264, 1996
13. Aviles-Santa L, Sinding J, Raskin P: Effects of metformin in patients with poorly controlled, insulin- treated type 2 diabetes mellitus: a randomized, double-blind, placebo-controlled trial. *Ann Intern Med* 131:182–188, 1999
14. Yki-Jarvinen H, Ryysy L, Nikkila K, Tulokas T, Vanamo R, Heikkila M: Comparison of bedtime insulin regimens in patients with type 2 diabetes mellitus: a randomized, controlled trial. *Ann Intern Med* 130:389–396, 1999

15. Robinson AC, Burke J, Robinson S, Johnston DG, Elkeles RS: The effects of metformin on glycemic control and serum lipids in insulin-treated NIDDM patients with suboptimal metabolic control. *Diabetes Care* 21:701–705, 1998
16. American Diabetes Association: Standards of medical care for patients with diabetes mellitus (Position Statement). *Diabetes Care* 22 (Suppl. 1):S32–S41, 1999
17. Cockcroft DW, Gault MH: Prediction of creatinine clearance from serum creatinine. *Nephron* 16:31–41, 1976
18. McQueen MJ: Clinical and analytical considerations in the utilization of cholinesterase measurements. *Clin Chim Acta* 237: 91–105, 1995
19. van Binsbergen JJ, Brouwer A, van Drenth BB, Haverkort ARM, Prins A, van der Weijden T: NHG-standaard cholesterol (Dutch). *Huisarts Wet* 34:551–557, 1991
20. van Binsbergen JJ, Grundmeyer HGLM, vanden Hoogen JPH: NHG-standaard hypertensie (Dutch). *Huisarts Wet* 34:551–557, 1991
21. Passing H, Bablok W: A new biometrical procedure for testing the equality of measurements from two different analytical methods. I. Application of linear regression procedures for method comparison studies in clinical chemistry. *J Clin Chem Clin Biochem* 21:709–720, 1983
22. Passing H, Bablok W: Comparison of several regression procedures for method comparison studies and determination of sample sizes. II. Application of linear regression procedures for method comparison studies in Clinical Chemistry. *J Clin Chem Clin Biochem* 22:431–445, 1984
23. Friedewald WT, Levy RI, Fredrickson DS: Estimation of the concentration of low-density lipoprotein cholesterol in plasma, without use of the preparative ultracentrifuge. *Clin Chem* 18:499–502, 1972
24. Frison L, Pocock SJ: Repeated measures in clinical trials: analysis using mean summary statistics and its implications for design. *Stat Med* 11:1685–704, 1992
25. Dunn C, Peters D: Metformin: a review of its pharmacological properties and therapeutic use in non-insulin-dependent diabetes mellitus. *Drugs* 49:721–749, 1995
26. Wiernsperger NF, Bailey CJ: The antihyperglycaemic effect of metformin: therapeutic and cellular mechanisms. *Drugs* 58 (Suppl. 1):31–39, 75–82, 1999
27. Relimpio F, Pumar A, Losada F, Mangas MA, Acosta D, Astorga R: Adding metformin versus insulin dose increase in insulin-treated but poorly controlled type 2 diabetes mellitus: an open-label randomized trial. *Diabet Med* 15:997–1002, 1998
28. Shinozaki K, Naritomi H, Shimizu T, Suzuki M, Ikebuchi M, Sawada T, Harano Y: Role of insulin resistance associated with compensatory hyperinsulinemia in ischemic stroke. *Stroke* 27:37–43, 1996
29. Despres JP, Lamarche B, Mauriege P, Cantin B, Dagenais GR, Moorjani S, Lupien PJ: Hyperinsulinemia as an independent risk factor for ischemic heart disease. *N Engl J Med* 334:952–957, 1996
30. DeFronzo RA, Ferrannini E: Insulin resistance: a multifaceted syndrome responsible for NIDDM, obesity, hypertension, dyslipidemia, and atherosclerotic cardiovascular disease. *Diabetes Care* 14:173–194, 1991
31. Taskinen MR, Kuusi T, Helve E, Nikkila EA, Yki-Jarvinen H: Insulin therapy induces antiatherogenic changes of serum lipoproteins in noninsulin-dependent diabetes. *Arteriosclerosis* 8:168–177, 1988
32. Pentikainen PJ, Voutilainen E, Aro A, Uusitupa M, Penttila I, Vapaatalo H: Cholesterol lowering effect of metformin in combined hyperlipidemia: placebo controlled double blind trial. *Ann Med* 22:307–312, 1990
33. Fontbonne A, Charles MA, Juhan-Vague I, Bard JM, Andre P, Isnard F, Cohen JM, Grandmottet P, Vague P, Safar ME, Eschwege E: The effect of metformin on the metabolic abnormalities associated with upper-body fat distribution: BIGPRO Study Group. *Diabetes Care* 19:920–926, 1996
34. Giugliano D, De Rosa N, Di Maro G, Marfella R, Acampora R, Buoninconti R, D'Onofrio F: Metformin improves glucose, lipid metabolism, and reduces blood pressure in hypertensive, obese women. *Diabetes Care* 16:1387–1390, 1993
35. Maziere JC, Maziere C, Mora L, Gardette J, Salmon S, Auclair M, Polonovski J: The antidiabetic drug metformin decreases cholesterol metabolism in cultured human fibroblasts. *Atherosclerosis* 71: 27–33, 1988
36. Scott L, Tomkin G: Changes in hepatic and intestinal cholesterol regulatory enzymes: the influence of metformin. *Biochem Pharmacol* 32:827–830, 1983
