# Supplementary material for: Metformin and N-terminal pro B-type natriuretic peptide in type 2 diabetes patients, a post-hoc analysis of a randomized controlled trial
Source: PLoS One. 2021 Apr 8;16(4):e0247939. doi: 10.1371/journal.pone.0247939 (PMC8031400; doi:10.1371/journal.pone.0247939)
Supplement: S2 File — (PDF) [file pone.0247939.s003.pdf]

Long-term Effects of Metformin on Metabolism and Microvascular and Macrovascular Disease in Patients With Type 2 Diabetes Mellitus

Adriaan Kooy, MD, PhD; Jolien de Jager, MD; Philippe Lehert, PhD; Daniël Bets, MSc; Michiel G. Wulffelé, MD, PhD; Ab J. M. Donker, MD, PhD; Coen D. A. Stehouwer, MD, PhD

Background: We investigated whether metformin hydrochloride has sustained beneficial metabolic and (cardio) vascular effects in patients with type 2 diabetes mellitus (DM2).

Methods: We studied 390 patients treated with insulin in the outpatient clinics of 3 hospitals in a randomized, placebo-controlled trial with a follow-up period of 4.3 years. Either metformin hydrochloride, 850 mg, or placebo (1-3 times daily) was added to insulin therapy. The primary end point was an aggregate of microvascular and macrovascular morbidity and mortality. The secondary end points were microvascular and macrovascular morbidity and mortality, as separate aggregate scores. In addition, effects on hemoglobin A_{1c} (HbA_{1c}), insulin requirement, lipid levels, blood pressure, body weight, and body mass index were analyzed.

Results: Metformin treatment prevented weight gain (mean weight gain, −3.07 kg [range, −3.85 to −2.28 kg]; $P < .001$), improved glycemic control (mean reduction in HbA_{1c} level, 0.4% percentage point [95% CI, 0.55-0.25]; $P < .001$) (where CI indicates confidence interval), despite the aim of similar glycemic control in both groups, and reduced insulin requirements (mean reduction, 19.63 IU/d [95% CI, 24.91-14.36 IU/d]; $P < .001$). Metformin was not associated with an improvement in the primary end point. It was, however, associated with an improvement in the secondary, macrovascular end point (hazard ratio, 0.61 (95% CI, 0.40-0.94; $P = .02$), which was partly explained by the difference in weight. The number needed to treat to prevent 1 macrovascular end point was 16.1 (95% CI, 9.2-66.6).

Conclusions: Metformin, added to insulin in patients with DM2, improved body weight, glycemic control, and insulin requirements but did not improve the primary end point. Metformin did, however, reduce the risk of macrovascular disease after a follow-up period of 4.3 years. These sustained beneficial effects support the policy to continue metformin treatment after the introduction of insulin in any patient with DM2, unless contraindicated.

Trial Registration: ClinicalTrials.gov Identifier: NCT00375388

Arch Intern Med. 2009;169(6):616-625

Author Affiliations: Department of Internal Medicine and Bethesda Diabetes Research Center, Bethesda Hospital, Hoogeveen, the Netherlands (Drs Kooy, de Jager, and Wulffelé); Department of Ophthalmology, Academic Medical Center, Amsterdam, the Netherlands (Dr de Jager); Department of Statistics, Faculty of Economics, Mons, Belgium (Dr Lehert); Clinical Research and Development, Amsterdam (Mr Bets); Department of Internal Medicine, Free University Medical Center, Amsterdam (Dr Donker); and Department of Internal Medicine, Maastricht University Medical Center, Maastricht, the Netherlands (Dr Stehouwer).

THE RISING INCIDENCE OF type 2 diabetes mellitus (DM2) makes it an increasingly important cause of cardiovascular disease (CVD) and death. Up to 75% of patients with DM2 will die from a cardiovascular complication.[1] Therefore, prevention of cardiovascular complications in patients with DM2 is crucial.

CME available online at www.jamaarchivescme.com and questions on page 541

Questions remain concerning the beneficial effects of metformin hydrochloride in patients with DM2. To our knowledge, the only randomized intervention trial on this subject was the UK Prospective Diabetes Study (UKPDS),[2] the results of which suggest a cardioprotective role for metformin. However, the design and analyses of the UKPDS have raised considerable debate.[2] Nevertheless, data from cohort studies support the results from the UKPDS,[3,4] but the need for clinical, randomized intervention trials still exists to help clarify this issue. In addition, the mechanisms through which metformin may decrease the risk of microvascular and macrovascular disease are unclear and may include reduction of weight gain and hyperinsulinemia, improvement of endothelial function and fibrinolysis, and reduction of low-grade inflammation, oxidative stress, and glycation.[5-7]

Type 2 diabetes mellitus is a progressive disease, and many patients will need treatment with insulin during the course

©2009 American Medical Association. All rights reserved.

of the disease.[8] Several short-term studies[9-11] in insulin-treated patients with DM2 have shown that metformin can improve glycemic control and reduce insulin requirements and weight gain. To our knowledge, the long-term beneficial effects of metformin in such patients have not been studied.

We hypothesized that, in patients with DM2 treated with insulin, metformin, compared with placebo, will have sustained beneficial metabolic effects, even at the same level of glycemic control, and thus decrease (cardio)vascular disease. We designed the randomized, placebo-controlled, multicenter trial "Hyperinsulinemia: the Outcome of its Metabolic Effects (HOME)" to investigate these issues during a planned follow-up period of 4.3 years.[11]

METHODS

PATIENTS

We included 390 patients with DM2 (age range, 30-80 years) as previously described.[11] All patients gave written informed consent. The medical ethical committees of the 3 participating hospitals approved the trial protocol. The trial was conducted in accordance with the Note for Guidance on Good Clinical Practice (CPMP/ICH/135/95) dated July 17, 1996, and in accordance with the Declaration of Helsinki (revised versions of Hong Kong in 1989 and Edinburgh, Scotland, in 2000).

STUDY DESIGN

The HOME Trial was conducted in the outpatient clinics of 3 hospitals (in the cities of Hoogeveen, Meppel, and Coevorden, the Netherlands). Patients were randomly allocated to either placebo or metformin by aid of a computer program, which allocated a random number to identical-looking boxes of either metformin or placebo. The trial design consisted of 3 phases, as shown in **Figure 1**.[11]

GLYCEMIC CONTROL AND INSULIN TITRATION

In the prerandomization phase, we aimed to optimize glycemic control by intensive glucose monitoring and insulin adjustments (target glucose levels, fasting: 72.1-126.1 mg/dL, and postprandial: 72.1-180.2 mg/dL). (To convert glucose to millimoles per liter, multiply by 0.0555.) All subjects monitored their glucose levels at home every 2 weeks (ie, just before and roughly 90 minutes after breakfast, lunch, and dinner, and at bedtime) using the same monitoring device (Glucotouch; Lifescan, Beerse, Belgium). Individual insulin titration took place according to good clinical practice to reach the target glucose levels and to prevent hypoglycemia. If the target values for glycemic control were difficult to reach, the study nurse consulted the principal investigator for advice on how to optimize the insulin therapy. This glucose-monitoring and insulin adjustment scheme was continued during the whole trial, as detailed elsewhere.[7,11]

CLINICAL END POINTS

The study was designed to examine the effects of metformin on metabolic and disease-related end points, as previously described[11] (**Table 1**). The metabolic end points examined were body weight, body mass index (BMI), calculated as weight in kilograms divided by height in meters squared), waist-to-hip ratio (WHR), hemoglobin A_{1c} (HbA_{1c}), home-monitored con-

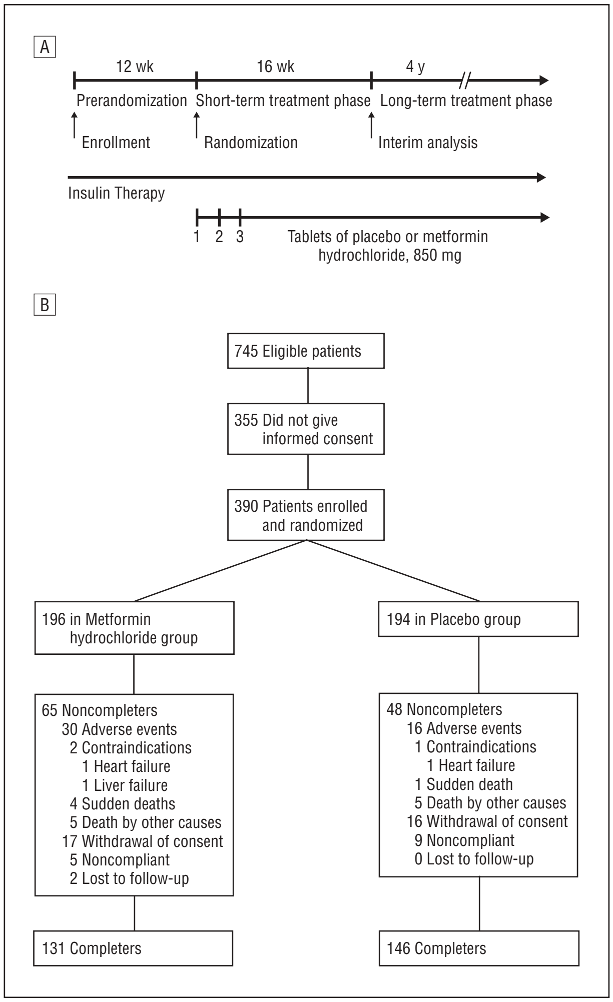

Figure 1. Trial schedule (A) and trial profile (B).

centrations of preprandial and postprandial glucose, fasting plasma insulin, and the daily dose of insulin. We also collected data on blood pressure (BP) and plasma lipids. In addition, we collected information on the incidence of hypoglycemic events. A hypoglycemic event was defined as a blood glucose concentration below 68.5 mg/dL with symptoms of hypoglycemia or a value below 54.1 mg/dL, with or without such symptoms. Data on weight, HbA_{1c}, home-monitored concentrations of preprandial and postprandial glucose levels, and BP were obtained at each visit (every 3 months). Waist-to-hip ratio was measured at baseline and after 4, 7, and 48 months. Plasma lipid levels were measured at baseline and every 6 months thereafter; plasma insulin level was measured at baseline, after 4 months, and every 12 months thereafter.

On the assumption that several shared determinants of macrovascular and microvascular disease (eg, hypertension, obesity, endothelial dysfunction, and chronic inflammation[13-15]) might be influenced by metformin,[5,7] the primary end point was constructed as a combination of macrovascular and microvascular disease. Secondary disease-related end points were macrovascular and microvascular disease separately (Table 1). Before disclosure of the treatment codes, an independent scientific committee (1 specialist in vascular medicine and 1 specialist in endocrinology) checked the registration, or absence thereof, of all disease-related end points. No misreporting was found.

To optimally study the metabolic end points, patients continued to participate in the trial after the occurrence of a nonfatal disease–related end point, unless a contraindication for

©2009 American Medical Association. All rights reserved.

Table 1. Metabolic and Disease-Related End Points of the HOME Trial[a]

Clinical Events	Definition
1. Myocardial infarction	Documented diagnosis by a cardiologist
2. Heart failure	Documented diagnosis by a cardiologist
3. Changes of ECG	Minnesota scores 1.1-1.3, 4.1-4.3, 5.1-5.3, 7.1
4. Acute coronary syndrome	Documented diagnosis by a cardiologist; having resulted in hospital admission
5. Diabetic foot	Documented diagnosis by an internist and/or surgeon; having resulted in hospital admission
6. Stroke	Documented diagnosis by a neurologist
7. Transient ischemic attack	Documented diagnosis by a neurologist
8. Peripheral arterial disease	Diagnosed by angiography
9. Peripheral arterial reconstruction	Determined by a physician and well documented in the original medical record and in the CRF
10. PTCA	Determined by a physician and well documented in the original medical record and in the CRF
11. CABG	Determined by a physician and well documented in the original medical record and in the CRF
12. Nontraumatic amputation	Determined by a physician and well documented in the original medical record and in the CRF
13. Sudden death	Determined by a physician and well documented in the original medical record and in the CRF
14. Progression of retinopathy	Classified by an ophthalmologist ($A \rightarrow C$ or $B \rightarrow C$), where A indicates none; B, nonproliferative; C, proliferative
15. Progression of nephropathy	$A \rightarrow B$ or $A \rightarrow C$ or $B \rightarrow C$; expressed as albuminuria (albumin to creatinine ratio in urine, A/C in milligrams per millimole), where A indicates normoalbuminuria; B, microalbuminuria; C, macroalbuminuria. Men were normal at A/C $<$ 2.5; had microalbuminuria at 2.5 $<$A/C $<$25, and had macroalbuminuria at $>$25. Women were normal at A/C $<$3.5, had microalbuminuria at 3.5 $<$A/C $<$35, and had macroalbuminuria at $>$35.
16. Progression of neuropathy ($A \rightarrow B$ or $A \rightarrow C$ or $A \rightarrow D$ or $B \rightarrow C$ or $B \rightarrow D$ or $C \rightarrow D$, with a difference in score of at least 6 points)	Diabetic polyneuropathy was evaluated by constructing a neuropathy score, where A indicates normal or 0 points; B, mild, 1-9 points; C, moderate, 10-18 points; D, severe, 19-33 points.[b]
17. Death by other cause	Noncardiovascular nonsudden death

Abbreviations: CABG, coronary artery bypass graft; CRF, clinical research file; ECG, electrocardiogram; HOME, Hyperinsulinemia: the Outcome of its Metabolic Effects; PTCA, percutaneous transluminal coronary angioplasty.

[a]The metabolic end points were lipid profile, body weight, body mass index, waist-to-hip ratio, plasma hemoglobin A_{1c} concentration, home-monitored concentrations of preprandial and postprandial glucose, plasma insulin concentration, daily dose of insulin, blood pressure, plasma lipids. The primary end points were clinical events 1 to 16, and secondary end points were as follows: (1) macrovascular: clinical events 1 to 13 and (2) microvascular: clinical events 14 to 16.

[b]See Valk et al.[12]

metformin emerged. Therefore, a patient who completed the planned 4.3-year follow-up period (a "completer") may or may not have encountered a disease-related end point. A noncompleter could thus have been withdrawn before the final visit for a variety of reasons: adverse effects, withdrawal of consent, loss to follow-up, a fatal end point, or because of the development of a contraindication to metformin, notably renal or heart failure (New York Heart Association class III/IV). We screened for renal failure by monitoring creatinine clearance. Patients with a creatinine clearance in the range of 40 to 60 mL/min/1.73 m^2 were allowed a maximum of two 850-mg tablets per day; those with a range of 30 to 40 mL/min/1.73 m^2 were allowed one 850-mg tablet per day; and those below 30 mL/min/1.73 m^2 were withdrawn from the study. (To convert creatinine clearance to milliliters per second per meters squared, multiply by 0.0167.)

STATISTICAL ANALYSES

Sample Size and Power Analysis

The planned study sample size of 390 patients was based on an expected difference in the occurrence of the primary end point of at least 8% points between the treatment groups after 4.3 years, with an expected incidence of 20% in the placebo group and 12% in the metformin group (1-tailed *t* test on proportional hazard regression with $\alpha=0.05$ and $\beta=0.25$).

Data Analysis

The data presented herein concern all randomized patients (intention-to-treat [ITT] sample; n=390); 95% confidence intervals (CIs) are used, and *P* values for the individual variables represent 2-sided *t* tests.

The effects of metformin on the metabolic end points were assessed by analyzing the complete time course for each variable. For this purpose, the summary mean (mean score of nonmissing values over the entire observation period) constitutes the appropriate repeated measurement technique.[16] This summary mean was then compared between the treatment groups through an analysis of covariance, while adjusting for baseline values and baseline differences in age, sex, smoking, and the prior occurrence and severity of CVD. The χ^2 test was used to compare the number of hypoglycemic events between the 2 treatment groups.

The effects of metformin on the disease-related end points were assessed by comparing time to first event by means of proportional hazards multiple regression analyses. This was the primary analysis for both the primary and the secondary end points. Because of the baseline differences in age, sex, smoking, statin use, and the prior occurrence and severity of CVD between the 2 treatment groups, we adjusted for these variables in all analyses. As an additional analysis, a multiple end point survival technique was used to take into account multiple events.[17] The severity of the cardiovascular history at baseline was computed as the sum score of cardiovascular events as follows: myocardial infarction absent=0, present=1; cardiovascular intervention (peripheral arterial reconstruction, percutaneous transluminal coronary angioplasty, and coronary artery bypass graft) absent=0, present=1; transient ischemic attack absent=0, present=1; stroke absent=0, present=1; dyspnea New York Heart Association class, no=0, I=1, II=2, III=3, IV=4; angina pectoris New York Heart Association, no=0, I=1, II=2, III=3, IV=4; intermittent claudication, no=0, more than 100 m=1, 50 to 100 m=2, less than 50 m=3, rest=4; and amputation absent=0, present=1.

To assess the extent to which the metformin-associated changes in the disease-related end points could be explained

©2009 American Medical Association. All rights reserved.

by the metformin-associated changes in the metabolic end points, we used proportional hazards multiple regression analysis to reanalyze the HRs after adjusting for metabolic variables. Main effects and first-order interaction with treatments were tested, and nonsignificant effects ($P < .05$) were sequentially removed to reduce type I errors and promote stability of the model.

RESULTS

PATIENTS

We screened the medical files of all 3 participating outpatient clinics and identified 745 eligible patients. All were approached to enroll into the trial, and 390 subjects gave written informed consent. A total of 196 subjects were randomized to receive metformin and 194 to receive placebo. Of the 390 included patients, 277 subjects (72%) completed the HOME Trial (completers). Of the noncompleters, 46 experienced adverse events (30 metformin, 16 placebo), 3 patients developed a contraindication to metformin (1 liver failure [metformin]; 2, heart failures [1 metformin, 1 placebo]), 15 encountered a fatal disease–related end point (9 metformin, 6 placebo), 47 withdrew their consent (22 metformin, 25 placebo), and 2 patients were lost to follow-up (2 metformin). Of the 46 patients with adverse events, 33 experienced diarrhea (22 metformin, 11 placebo); 20, flatulence (10 metformin, 10 placebo); 15, fatigue (7 metformin, 8 placebo); 14, pruritus (5 metformin, 9 placebo); 15, headaches (6 metformin, 9 placebo); 16, heartburn (7 metformin, 9 placebo); and 20, nausea (10 metformin, 10 placebo). Noncompleters did not differ from completers with respect to duration of DM2, prior occurrence and severity of CVD, age, or weight. More women than men were noncompleters (male to female ratio, 1:1.8 metformin vs 1:1 placebo; $P = .01$).

Table 2 shows baseline characteristics of all randomized patients. Patients randomized to metformin were slightly older than patients randomized to placebo (mean [SD] age, 63.6 [9.6] vs 59.1 [11.0] years, respectively), had a more extensive cardiovascular history (1.17 vs 0.92, respectively) and were less often smokers. The other characteristics were comparable between the treatment groups. The actual mean daily doses in the metformin group in the short- and long-term treatment phases were 2163 mg and 2050 mg, respectively. Although the use of statins was low at baseline, in accordance with treatment guidelines at the start of the study, its use increased to 67 patients in the metformin group and 54 patients in the placebo group at the final visit.

Table 2. Baseline Characteristics of the Patients (Intention-to-Treat Sample)[a]

Characteristic	Placebo Group (n=194)	Metformin Hydrochloride Group (n=196)
Demography		
Men/women	97/97	81/115
Age, mean (SD), y	59 (11)	64 (10)
Currently smoking	59 (30)	38 (19)
Duration of type 2 diabetes mellitus, y	12 (8)	14 (9)
Duration of insulin treatment, y	6 (6)	7 (8)
Concomitant medication		
Lipid-lowering drugs, No. (%)	31 (16)	32 (16)
BP-lowering drugs, No. (%)	75 (39)	93 (47)
Metabolic variables		
Weight, kg	87 (15)	85 (16)
BMI	30 (5)	30 (5)
Waist-to-hip ratio		
Men	1.03 (0.1)	1.02 (0.1)
Women	0.93 (0.1)	0.92 (0.1)
Plasma HbA_{1c} level, %	7.9 (1.2)	7.9 (1.2)
Preprandial glucose level, mg/dL	158.6 (32.4)	155.0 (32.4)
Postprandial glucose level, mg/dL	183.8 (36.0)	183.8 (37.8)
Serum insulin, μIU/mL	43.3	35.7
Daily dose of insulin, IU/d	64 (25)	62 (29)
Systolic BP, mm Hg	159 (25)	160 (25)
Diastolic BP, mm Hg	86 (11)	86 (12)
Total cholesterol level, mg/dL	212.4 (46.3)	216 (50.2)
LDL cholesterol level, mg/dL	131.3 (38.6)	139.0 (42.5)
Triglyceride level, mg/dL	168.1 (132.7)	150.4 (106.2)
HDL cholesterol level, mg/dL	50.2 (15.4)	50.2 (15.4)
Prior macrovascular and microvascular disease		
Cardiovascular history	0.92 (1.3)	1.17 (1.4)
Diabetic polyneuropathy score	7.51 (5.4)	8.36 (6.3)

Abbreviations: BMI, body mass index (calculated as weight in kilograms divided by height in meters squared); BP, blood pressure; HbA_{1c}, hemoglobin A_{1c}; HDL, high-density lipoprotein; LDL, low-density lipoprotein.

SI conversion factors. To convert serum glucose to millimoles per liter, multiply by 0.0555; to convert plasma insulin to picomoles per liter, multiply by 6.945; to convert plasma HbA_{1c} to a proportion of total hemoglobin, multiply by 0.01; to convert total, HDL, and LDL cholesterol to millimoles per liter, multiply by 0.0259; to convert triglycerides to millimoles per liter, multiply by 0.0113.

[a] Data are given as mean (SD) except where indicated.

METABOLIC END POINTS

Improved Glycemic Control and Reduced Insulin Requirements With Metformin

Despite the aim of similar glycemic control in both groups, after 4.3 years of treatment, the mean difference in the summary mean for HbA_{1c} between the metformin and placebo groups was −0.40 percentage point (−0.55 to −0.25; $P < .001$) (to convert HbA_{1c} to a proportion of total hemoglobin, multiply by 0.01). For home-monitored concentrations of preprandial and postprandial glucose, the mean differences were −5.2 mg/dL (range, −9.2 to −1.3 mg/dL; $P = .01$) and −7.9 mg/dL (range, −12.3 to −3.6 mg/dL; $P < .01$), respectively. For plasma insulin, the mean difference was −17.85 μIU/mL (range, −33.26 to −2.30 μIU/mL; $P = .02$) (to convert serum insulin to picomoles per liter, multiply by 6.945). For the daily dose of insulin, the mean difference was −19.63 IU/d (range, −24.91 to −14.36 IU/d; $P < .001$) or −0.18 IU/kg (range, −0.23 to −0.12 IU/d; $P < .001$) (**Table 3** and **Figure 2** and **Figure 3**).

Use of Metformin to Prevent Weight Gain

After 4.3 years of treatment, the mean difference in the summary mean for body weight between the metformin and placebo groups was −3.07 kg (range, −3.85 to −2.28 kg; $P < .001$). The mean difference for BMI was −1.09 (95% CI, −1.37 to −0.81; $P < .001$). The mean difference for the WHR was −0.015 (95% CI, −0.029 to −0.001; $P = .04$) (Figure 3).

©2009 American Medical Association. All rights reserved.

Table 3. Overview of the Main End Points: Metabolic End Points[a]

End Point	Placebo Group (n=194) Baseline	Last Visit	Summary Mean	Metformin Hydrochloride Group (n=196) Baseline	Last Visit	Summary Mean
Body weight, kg	87 (15)	91 (17)	90 (16)	85 (16)	87 (17)	85 (16)
BMI	30 (5)	31 (5)	31 (5)	30 (5)	30 (5)	30 (5)
Waist-to-hip ratio						
Men	1.03 (0.1)	1.03 (0.1)	1.03 (0.1)	1.02 (0.1)	1.03 (0.1)	1.03 (0.1)
Women	0.93 (0.1)	0.95 (0.1)	0.94 (0.1)	0.92 (0.1)	0.93 (0.1)	0.93 (0.1)
Plasma HbA_{1C} level, %	7.9 (1.2)	7.9 (1.1)	7.9 (1.0)	7.9 (1.2)	7.7 (1.1)	7.5 (1.0)
Preprandial glucose level, mg/dL	158.6 (32.4)	144.1 (27.0)	147.7 (28.8)	155.0 (32.4)	146.0 (28.8)	140.5 (23.4)
Postprandial glucose level, mg/dL	183.8 (36.0)	162.2 (28.8)	169.4 (23.4)	183.8 (37.8)	160.3 (34.3)	160.3 (27.0)
Serum insulin level, μIU/mL	43.3 (98.8)	75.2 (153.8)	79.0 (138.4)	35.7 (78.5)	46.5 (86.1)	45.2 (92.3)
Daily dose of insulin, IU/d	64 (25)	100 (59)	84 (40)	62 (29)	75 (50)	67 (40)
Systolic BP, mm Hg	159 (25)	141 (14)	154 (17)	160 (25)	141 (18)	153 (18)
Diastolic BP, mm Hg	86 (11)	79 (10)	85 (9)	86 (12)	77 (11)	85 (9)
Plasma total cholesterol level, mg/dL	212.4 (50.2)	162.2 (30.9)	181.5 (34.7)	216.2 (50.2)	162.2 (27.0)	185.3 (42.5)
Plasma LDL cholesterol level, mg/dL	131.3 (38.6)	84.9 (23.2)	104.2 (30.9)	139.0 (42.5)	81.1 (23.2)	108.1 (34.7)
Plasma triglyceride level, mg/dL	168.1 (132.7)	141.6 (141.6)	150.4 (106.2)	150.4 (106.2)	132.7 (79.6)	141.6 (88.5)
Plasma HDL cholesterol level, mg/dL	48.3 (15.4)	51.4 (15.4)	49.0 (15.4)	50.2 (15.4)	52.1 (15.4)	51.0 (15.4)

Abbreviations: BMI, body mass index (calculated as weight in kilograms divided by height in meters squared); BP, blood pressure; HbA_{1C}, hemoglobin A_{1C}; HDL, high-density lipoprotein; LDL, low-density lipoprotein.

SI conversion factors. To convert serum glucose to millimoles per liter, multiply by 0.0555; to convert plasma HbA_{1c} to a proportion of total hemoglobin, multiply by 0.01; to convert serum insulin to picomoles per liter, multiply by 6.945; to convert total, HDL, and LDL cholesterol to millimoles per liter, multiply by 0.0259; to convert triglycerides to millimoles per liter, multiply by 0.0113.

[a] Data are given as mean (SD).

BP AND LIPID PROFILE

No Decrease in BP With Metformin

After 4.3 years of treatment, the mean difference in the summary mean between the metformin and placebo groups was −0.51 mm Hg (range, −2.76 to 1.44 mm Hg; *P*=.38) for systolic BP and −0.88 mm Hg (−3.21 to 1.45 mm Hg; *P*=.46) for diastolic BP.

No Improvement in Plasma Lipid Profile With Metformin

After 4.3 years of treatment, the difference in the summary mean between the metformin and placebo groups was −0.77 mg/dL (range, −4.63 to 6.18 mg/dL; *P*=.78) for total cholesterol level; 0.00 mg/dL (−4.63 to 4.63 mg/dL; *P*=.98) for low-density lipoprotein cholesterol level; 0.88 mg/dL (−9.73 to 12.39; *P*=.82) for triglyceride level; and 0.00 mg/dL (−123.9 to 56.0 mg/dL; *P*=.91) for high-density lipoprotein cholesterol level. The use of lipid-lowering drugs did not significantly differ between the groups (*P*=.45), and adjustment for use of lipid-lowering drugs did not change the results. (To convert total, low-density, high-density cholesterol to millimoles per liter, multiply by 0.0259; to convert triglycerides to millimoles per liter, multiply by 0.0113.)

HYPOGLYCEMIC EVENTS

After 4.3 years of treatment, no difference in the number of hypoglycemic events between the metformin and placebo groups existed (metformin, 2.1 events vs placebo, 2.6 total hypoglycemic events per person per year; *P*=.89). In 0.3 and 0.3 events per person per year, respectively, help from others was needed (*P*=.33).

DISEASE-RELATED END POINTS

No Decrease in the Risk of the Primary End Point With Metformin

The unadjusted event rates were 28% for patients in the placebo group and 31% in the metformin group. After adjustment for age, sex, smoking, and cardiovascular history, the HR for the primary end point was 0.92 (95% CI, 0.72-1.18; *P*=.33), and, if combined with death by other causes, 0.94 (95% CI, 0.74-1.19; *P*=.37) (**Table 4** and **Figure 4**).

Decrease in the Risk of the Secondary, Macrovascular End Point With Metformin

The unadjusted event rates were 18% for patients in the placebo group and 15% in the metformin group. After adjustment for age, sex, smoking, and cardiovascular history, the HR for the secondary, macrovascular end point was 0.60 (95% CI, 0.40-0.92; *P*=.04). The absolute risk difference between the groups was – 6.1% (95% CI, −10.5 to −1.5%; *P*=.04), resulting in a number needed to treat (NNT) of 16 (95% CI, 9-67) to prevent 1 macrovascular end point. Exclusion of sudden death from the secondary, macrovascular end point did not change the results; HR, 0.61 (95% CI, 0.40-0.94; *P*=.02). Figure 4 shows the survival functions for the 2 treatment groups.

No Decrease in the Risk of the Secondary, Microvascular End Point With Metformin

The unadjusted event rates were 15% for patients in the placebo group and 17% in the metformin group. After adjustment for age, sex, smoking, and prior diabetic poly-

©2009 American Medical Association. All rights reserved.

neuropathy, the HR for the secondary, microvascular end point was 1.04 (95% CI, 0.75-1.44; $P = .43$).

ADDITIONAL ANALYSES

After adjusting for the change in weight, the HR for the secondary, macrovascular end point was 0.77 (95% CI, 0.55-1.09; $P = .33$) compared with 0.60 (95% CI, 0.40-0.92; $P = .04$) without adjusting for change in weight. However, adjustments for the metformin-associated changes in other metabolic efficacy variables, such as BP or lipid profile, did not materially change our results. For example, after adjusting for changes in HbA_{1c} level, daily dose of insulin, and systolic BP, the HR for the secondary, macrovascular end point was 0.34 (95% CI, 0.21-0.56; $P = .001$) compared with 0.60 (95% CI, 0.40-0.92; $P = .04$) without such adjustments. The HR for the secondary macrovascular end point without including diabetic foot was 0.60 (95% CI, 0.44-0.85; $P = .05$) compared with 0.60 (95% CI, 0.40-0.92; $P = .04$). There was no interaction between hospital center and treatment, and adjustment for treatment center did not change any of the results. Analyses using the per protocol population showed similar results, although slightly more in favor of metformin, compared with the ITT population. Analysis of the metabolic end points using the last observation carried forward instead of the summary mean yielded very similar results.

There was loss of glycemic control over time (Figure 2), which was similar in both groups. In the placebo and metformin groups, 14% and 16% of patients, respectively, had an HbA_{1c} level of less than 7% at the final visit, and in both groups 14% of patients had an HbA_{1c} level higher than 8% at the final visit while having had an HbA_{1c} lower than 7% at baseline. Conversely, 10% and 9% of patients in the placebo and metformin groups, respectively, had an HbA_{1c} level lower than 7% at the final visit, while having had an HbA_{1c} level higher than 8% at baseline.

COMMENT

Our study on the effects of long-term metformin treatment in patients with DM2 treated with insulin had 2 main findings. First, metformin treatment was associated with beneficial effects on outcomes such as body weight and insulin requirements and with moderately beneficial effects on glycemic control (despite the aim of similar glycemic control in both groups), but not on BP and the plasma lipid profile. Second, metformin treatment did not decrease the risk of the primary end point but did decrease the risk of the secondary, macrovascular end point.

The favorable effects of metformin treatment on weight gain and insulin requirements are in accordance with previous findings from short-term studies.[9-11] The absence of a BP-lowering effect of metformin is also consistent with previous studies and meta-analyses.[18-21] Previous short-term studies on plasma lipid levels have shown either no benefit or only a small effect of metformin treatment.[19,21,22] Importantly, our study shows that the beneficial effects on weight gain and insulin requirements persisted during 4.3 years of follow-up. The reductions

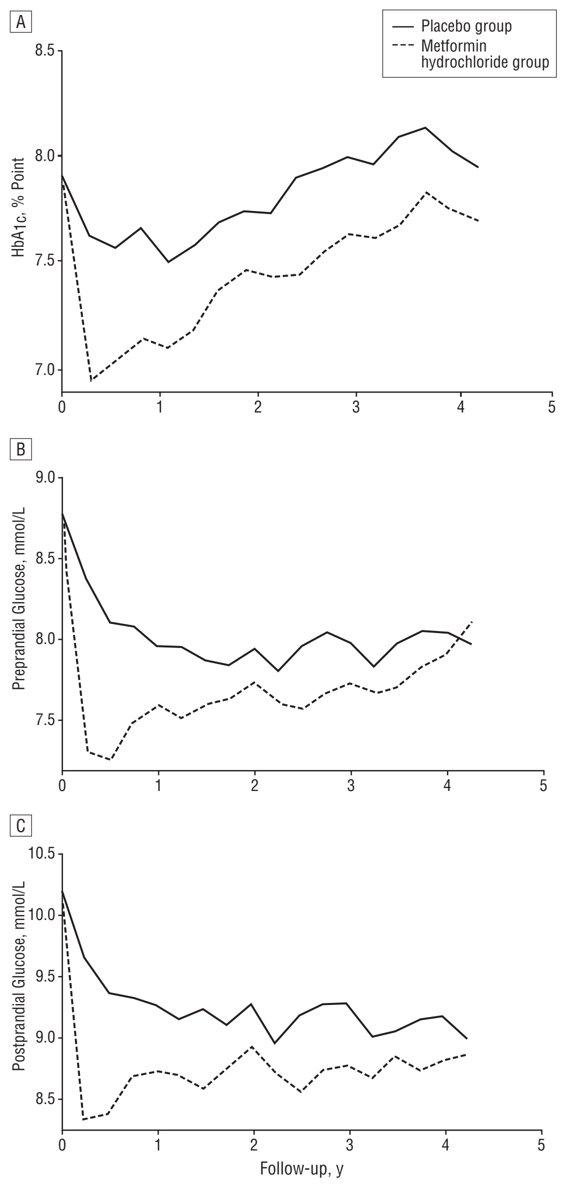

Figure 2. Glycemic control: hemoglobin A_{1c} (HbA_{1c}) and preprandial and postprandial glucose values. Despite the aim of similar glycemic control in both groups, the summary means were significantly different between the groups for HbA_{1c} ($P < .001$) (A), home-monitored preprandial glucose ($P = .01$) (B), and home-monitored postprandial glucose ($P < .01$) (C). To convert serum glucose from millimoles per liter to milligrams per deciliter, divide by 0.0555.

of the daily dose of insulin and of plasma insulin levels seem to indicate lower insulin exposure levels in the metformin group.

The beneficial effects of metformin on weight and on insulin requirements continued to improve during 4.3 years of treatment. The improvements in glycemic control, however, occurred rapidly but were not maintained throughout the long treatment period. The rate of the loss of glycemic control (approximately 1 percentage point increase in HbA_{1c} level over 5 years) was similar to that seen in the UKPDS. It is unclear why this loss of glycemic control, which was comparable between the

©2009 American Medical Association. All rights reserved.

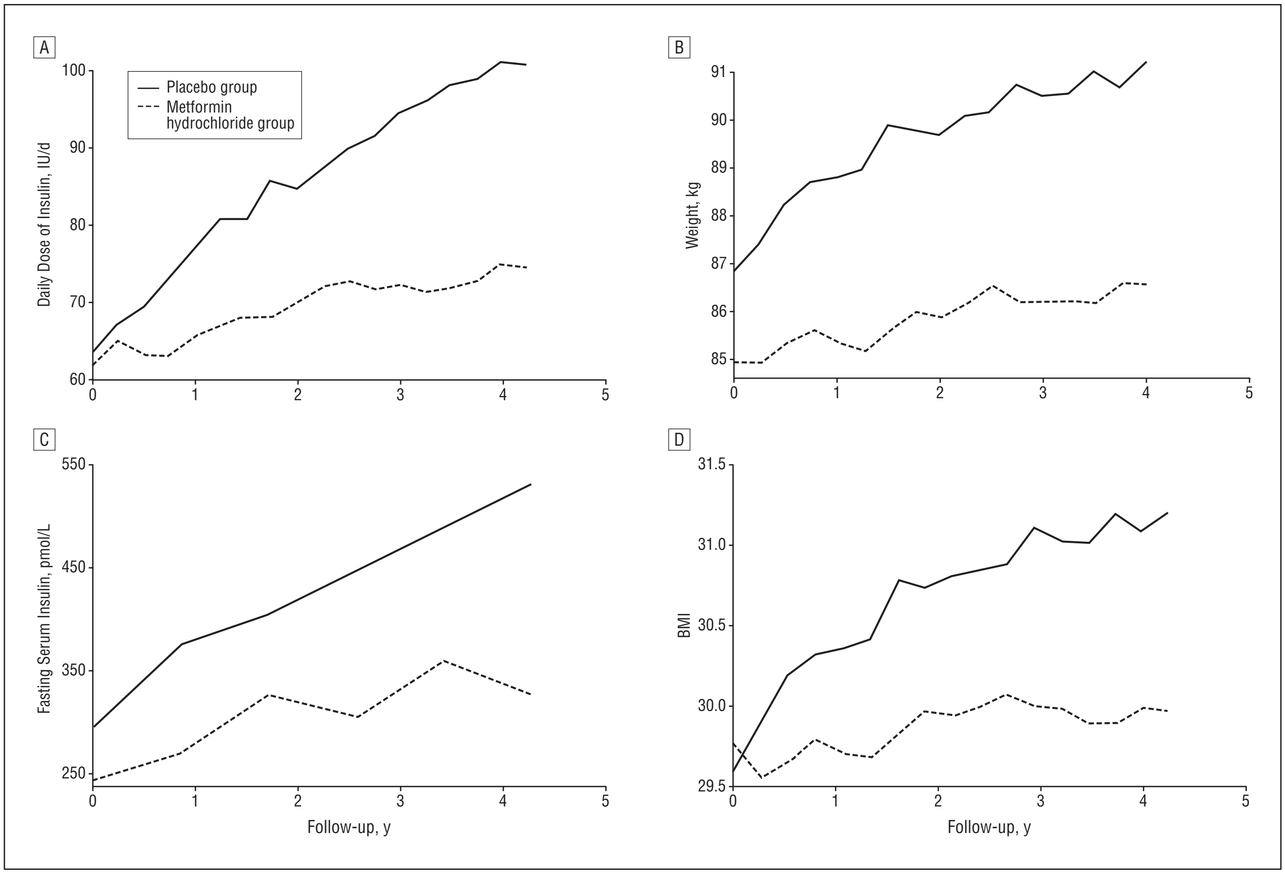

Figure 3. Insulin requirements and weight. The summary means were significantly different between the groups for A, daily dose of insulin ($P < .001$); B, body weight ($P < .001$); C, fasting plasma insulin ($P = .02$); and D, body mass index (BMI) (calculated as weight in kilograms divided by height in meters squared) ($P < .001$). To convert serum insulin from picomoles per liter to micro–international units per milliliter, divide by 6.945.

Table 4. Overview of the Main End Points: Disease-Related End Points[a]

	Placebo Group (n=194)		Metformin Hydrochloride Group (n=196)	
**End Point**	**Baseline**	**Last Visit**	**Baseline**	**Last Visit**
Myocardial infarction	21 (11)	25 (13)	24 (12)	28 (14)
Heart failure	0	4 (2)	0	3 (2)
Ischemic changes of ECG	NA	14 (7)	NA	10 (5)
Acute coronary syndrome	0	7 (4)	4 (2)	6 (3)
Diabetic foot	6 (3)	11 (6)	6 (3)	9 (5)
Stroke	8 (4)	9 (5)	8 (4)	9 (5)
Transient ischemic attack	10 (5)	12 (6)	8 (4)	10 (5)
Peripheral arterial disease	10 (5)	18 (9)	14 (7)	21 (11)
Cardiovascular intervention	18 (9)	27 (14)	27 (14)	34 (17)
Nontraumatic amputation	3 (2)	4 (2)	5 (3)	7 (4)
Sudden death	NA	1 (1)[b]	NA	4 (2)[c]
Progression of retinopathy	NA	0	NA	1 (1)
Progression of nephropathy	NA	14 (7)	NA	15 (8)
Progression of neuropathy	NA	18 (9)	NA	19 (10)
Death by other causes	NA	5 (3)	NA	5 (3)

Abbreviations: ECG, electrocardiogram; NA, not applicable.
[a]Data are given as number (percentage). Differences (with *P* values) between metformin and placebo (Figures 3 and 4) are described in more detail in the "Disease-Related End Points" subsection in the "Results" section.
[b]Cardiovascular death.
[c]Three cardiovascular deaths and 1 fatal car crash.

treatment groups, occurred. Insulin adjustments were made based on a treatment protocol, using target glucose values. Glycemic control deterioration after initial improvement has been frequently described before; a higher risk of glycemic relapse has been associated with insulin treatment, longer duration of DM2, and weight

©2009 American Medical Association. All rights reserved.

gain.[23] In addition, glycemic relapse has been, at least partly, attributed to the progressive nature of DM2.[24] Indeed, in our study, higher insulin dosages were needed over time, especially in the placebo group. A reluctance of both physician and patient to increase the insulin dosage in the face of deteriorating glycemic control has been described as a strong contributor to the deterioration of glycemic control.[25,26] Our study nurses, however, carefully performed the protocol for glycemic control during the whole period of follow-up.

Overall, glycemic control was better in the metformin group, despite the aim of similar glycemic control in both groups. These data suggest that, in patients with DM2 treated with insulin, metformin may affect glucose metabolism by improving the hepatic responsiveness to insulin and by increasing the release of glucagon-like peptide type 1.[27,28]

To our knowledge, no randomized, placebo-controlled trials on the effects of metformin on macrovascular or microvascular disease in patients with insulin-treated DM2 have been reported. In our study, metformin treatment was associated only with favorable effects on macrovascular disease but, somewhat unexpectedly, not on microvascular disease. Several reasons may account for this. In studies that have shown improved microvascular outcomes in treated patients with DM2, larger HbA_{1c} differences were maintained over longer periods of time.[24,29] In addition, improvements in BP have been shown to be an important contributor to a decrease in microvascular disease risk.[30,31] Thus, the small effect of metformin on glycemic control and the lack of an effect on BP may explain why microvascular outcomes were not improved within the follow-up period of 4.3 years. The absence of a treatment effect on microvascular disease may explain why significant changes were not observed in the primary end point ($P = .33$), in which microvascular events were incorporated.

In our study, the favorable effects of metformin on macrovascular disease could be partly explained (roughly 40%) by the metformin-associated change in weight. All other metformin-associated changes in metabolic or hemodynamic variables, such as HbA_{1C} level and daily dose of insulin, did not seem to contribute to the favorable effect of metformin on macrovascular disease. Taken together, these results suggest that metformin affects CVD partly by reducing weight, but that mechanisms other than improving glycemic control or reducing insulin requirements may be of importance as well. Previous studies have shown metformin to improve endothelial function and fibrinolysis,[5,32] independent of glycemic control, insulin requirements, or weight gain.[7] Endothelial dysfunction, in turn, has been strongly associated with an increased risk of CVD.[14,33-35] Metformin-associated improvements in endothelial function, however, were small.[6,7,32] Other possible mechanisms may include effects on advanced glycation end product levels or the secretion of adipocyte-derived mediators (eg, free fatty acids, leptin, resistin, and adiponectin).[6,36,37] These possible mechanisms require further investigation.

Strengths of our study include its randomized, placebo-controlled, double-blind design, its long follow-up period of 4.3 years, and finally the sustained participation

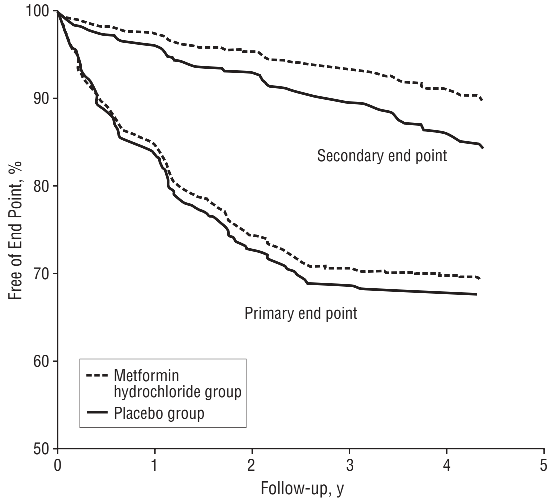

Figure 4. Survival functions for the primary (lower pair of curves) and the secondary, macrovascular (upper pair of curves) end points. Metformin treatment was not associated with an improvement in the primary end point. It was, however, associated with a decreased risk of the secondary, macrovascular end point (hazard ratio, 0.61 [95% confidence interval, 0.40-0.94; $P = .02$]). The number needed to treat to prevent 1 macrovascular end point was 16 (95% confidence interval, 9-67).

of patients in the trial after the occurrence of a nonfatal disease–related end point, thereby reducing dropout bias. In addition, the results were consistent across the different statistical analyses used.

Our study has several limitations. First, its relatively small sample size and consequently limited power may have obscured smaller treatment effects. To increase the power of our study, disease-related end points were constructed by combining separate clinical events regarding microvascular and macrovascular disease. An assumption in the construction of these disease-related end points is that its components are equally important, which is not necessarily true. In addition, the hypothesis that metformin influences both microvascular and macrovascular disease through shared underlying pathophysiologic characteristics, in the way, for instance, that obesity does, may not be correct. Diabetic foot was included in the primary end point, a combination of microvascular and macrovascular disease, as well as in the secondary, macrovascular end point. The etiology of diabetic foot lesions is a complex issue, but it is recognized that 3 key elements are involved: neuropathy, peripheral vascular disease, and infection.[38,39] Five of the 8 patients who developed a diabetic foot showed marked peripheral vascular disease on angiographic examination during their hospital admission. However, 3 patients had no angiographic documentation and could have developed a diabetic foot without any peripheral arterial disease. However, the HR for the secondary macrovascular end point without including diabetic foot was 0.60 (95% CI, 0.44-0.85; $P = .05$) compared with 0.60 (95% CI, 0.40-0.92; $P = .04$). Second, there was an imbalance between the 2 treatment groups after randomization. We adjusted for this in all analyses. To adjust for the difference in prior CVD, we constructed a way of measuring cardiovascu-

©2009 American Medical Association. All rights reserved.

lar history, which might not optimally reflect the medical history and severity of CVD at baseline. Therefore, the results, especially with regard to the secondary, macrovascular end point, must be interpreted with caution. Third, although all patients were treated in nonacademic hospitals, they did receive more intensive care than normally available in such centers, and our results may therefore not be generalizable to patients in other settings. Finally, we have conducted multiple analyses, and we cannot entirely exclude the possibility that the positive finding on the secondary end point is due to chance. However, several statistical approaches showed this to be a consistent finding.

In conclusion, we showed that in patients with DM2 treated with insulin, the addition of metformin resulted in improvements in weight, glycemic control, and insulin requirements, which were maintained after 4.3 years of treatment. However, metformin treatment did not reduce the risk of the primary end point. It may, however, reduce the risk of macrovascular disease, independently of reducing hyperinsulinemia, but partly related to the prevention of weight gain during insulin treatment. In general practice, when, owing to the progressive nature of DM2, insulin treatment is required, patients may benefit if metformin treatment is continued.

Accepted for Publication: November 3, 2008.
Correspondence: Adriaan Kooy, MD, PhD, Department of Internal Medicine and Bethesda Diabetes Center, Bethesda Diabetes Research Center, Mailbox 30.000, 7900 RA Hoogeveen, the Netherlands (kooy.a@bethesda.nl).
Author Contributions: Drs Kooy and de Jager contributed equally to this publication and contributed equally as primary coauthors. Drs Kooy, de Jager, and Lehert had full access to all of the data in the study and take responsibility for the integrity of the data and the accuracy of the data analysis. *Study concept and design:* Kooy, Donker, and Stehouwer. *Acquisition of data:* Kooy, de Jager, Bets, Wulffelé, and Stehouwer. *Analysis and interpretation of data:* Kooy, de Jager, Lehert, Wulffelé, Donker, and Stehouwer. *Drafting of the manuscript:* Kooy, de Jager, Wulffelé, and Stehouwer. *Critical revision of the manuscript for important intellectual content:* Kooy, Lehert, Bets, Wulffelé, and Donker. *Statistical analysis:* Kooy, de Jager, Lehert and Stehouwer. *Obtained funding:* Kooy. *Administrative, technical, and material support:* de Jager and Bets. *Study supervision:* Kooy, Lehert, and Stehouwer.
Financial Disclosure: None reported.
Funding/Support: The HOME Trial was supported by grants from Altana; Lifescan; E. Merck/Santé; Merck, Sharpe, & Dohme; and Novo Nordisk.
Role of the Sponsors: The sponsors had no role in the design and conduct of the study; in the collection, analysis, and interpretation of the data; or in the preparation, review, or approval of the manuscript.
Additional Contributions: We thank the study nurses Liesbeth Breedland, RN, and Els van Driesum, RN, for their dedication to the concerns of the patients and the quality of the treatments; Jan van der Kolk, MSc, Steven Berends, MSc, and Joop Verburg, MSc, for their technical assistance and validation of the laboratory techniques; Gerard de Groot, PhD, Rob Hoorn, MSc, and Erk Pieterse, MSc, for their hospitality at their laboratories; the independent scientific committee (Don Poldermans, MD, PhD, internist and professor of vascular medicine, and Henk Asscheman, MD, PhD, endocrinologist) for checking the registration or nonregistration of all disease-related end points; and all other members of the HOME Study group for their contribution to the HOME Trial.

REFERENCES

1. Bonow RO, Gheorghiade M. The diabetes epidemic: a national and global crisis. *Am J Med*. 2004;116(suppl 5A):2S-10S.
2. U.K. Prospective Diabetes Study (UKPDS) Group. Effect of intensive blood-glucose control with metformin on complications in overweight patients with type 2 diabetes (UKPDS 34). *Lancet*. 1998;352(9131):854-865.
3. Evans JMM, Ogston SA, Emslie-Smith A, Morris AD. Risk of mortality and adverse cardiovascular outcomes in type 2 diabetes: a comparison of patients treated with sulfonylureas and metformin. *Diabetologia*. 2006;49(5):930-936.
4. Johnson JA, Simpson SH, Toth WL, Majumdar SR. Reduced cardiovascular morbidity and mortality associated with metformin use in subjects with type 2 diabetes. *Diabet Med*. 2005;22(4):497-502.
5. Kirpichnikov D, McFarlane SI, Sowers JR. Metformin: an update. *Ann Intern Med*. 2002;137(1):25-33.
6. Beisswenger PJ, Howell SK, Touchette AD, Lal S, Swerzgold BS. Metformin reduces systemic methylglyoxal levels in type 2 diabetes. *Diabetes*. 1999;48(1):198-202.
7. De Jager J, Kooy A, Lehert P, et al. Effects of short-term treatment with metformin on markers of endothelial function and inflammatory activity in type 2 diabetes mellitus: a randomised, placebo-controlled trial. *J Intern Med*. 2005;257(1):100-109.
8. Marre M. Before oral agents fail: the case for starting insulin early. *Int J Obes Relat Metab Disord*. 2002;26(suppl 3):S25-S30.
9. Avilés-Santa L, Sinding J, Raskin P. Effects of metformin in patients with poorly controlled insulin-treated type 2 diabetes mellitus: a randomized, double-blind, placebo-controlled trial. *Ann Intern Med*. 1999;131(3):182-188.
10. Yki-Järvinen H, Ryysy L, Nikkilä K, Tulokas T, Vanamo R, Heikkilä M. Comparison of bedtime insulin regimens in patients with type 2 diabetes mellitus: a randomized, controlled trial. *Ann Intern Med*. 1999;130(5):389-396.
11. Wulffelé MG, Kooy A, Lehert P, et al. Combination of insulin and metformin in the treatment of type 2 diabetes. *Diabetes Care*. 2002;25(12):2133-2140.
12. Valk GD, Nauta JJP, Strijers RLM, Bertelsmann FW. Clinical examination versus neurophysiological examination in the diagnosis of diabetic polyneuropathy. *Diabet Med*. 1992;9(8):716-721.
13. Bonadonna RC, Cucinotta D, Fedele D, Riccardi G, Tiengo A; Metascreen Writing Committee. The metabolic syndrome is a risk indicator of microvascular and macrovascular complications in diabetes: results from Metascreen, a multicenter diabetes clinic-based survey. *Diabetes Care*. 2006;29(12):2701-2707.
14. de Jager J, Dekker JM, Kooy A, et al. Endothelial dysfunction and low-grade inflammation explain much of the excess cardiovascular mortality in individuals with type 2 diabetes: the Hoorn study. *Arterioscler Thromb Vasc Biol*. 2006;26(5):1086-1093.
15. Nguyen TT, Wong TY. Retinal vascular manifestations of metabolic disorders. *Trends Endocrinol Metab*. 2006;17(7):262-268.
16. Frison L, Pocock SJ. Repeated measures in clinical trials: analysis using mean summary statistics and its implications for design. *Stat Med*. 1992;11(13):1685-1704.
17. Qiou Z, Ravishanker N, Dey DK. Multivariate survival analysis with positive stable frailties. *Biometrics*. 1999;55(2):637-644.
18. Wulffelé MG, Kooy A, Lehert P, Bets D, Donker AJM, Stehouwer CDA. Does metformin decrease blood pressure in patients with type 2 diabetes intensively treated with insulin? *Diabet Med*. 2005;22(7):907-913.
19. Granberry MC, Fonseca VA. Cardiovascular risk factors associated with insulin resistance: effects of oral antidiabetic agents. *Am J Cardiovasc Drugs*. 2005;5(3):201-209.
20. Schäfers RF. Do effects on blood pressure contribute to improved clinical outcomes with metformin? *Diabetes Metab*. 2003;29(4, pt 2):6S62-6S70.
21. Wulffelé MG, Kooy A, de Zeeuw D, Stehouwer CDA, Gansevoort RT. The effect of metformin on blood pressure, plasma cholesterol, and triglycerides in type 2 diabetes mellitus: a systematic review. *J Intern Med*. 2004;256(1):1-14.
22. Rodríguez-Moctezuma JR, Robles-Lopez G, Lopez-Carmona JM, Gutierrez-Rosas MJ. Effects of metformin on the body composition in subjects with risk factors for type 2 diabetes. *Diabetes Obes Metab*. 2005;7(2):189-192.

©2009 American Medical Association. All rights reserved.

23. Graber AL, Shintani AK, Wolff K, Brown A, Elasy TA. Glycemic relapse in type 2 diabetes. *Endocr Pract*. 2006;12(2):145-151.
24. UK Prospective Diabetes Study (UKPDS) Group. Intensive blood-glucose control with sulphonylureas or insulin compared with conventional treatment and risk of complications in patients with type 2 diabetes (UKPDS 33). *Lancet*. 1998;352(9131):837-853.
25. Phillips LS, Branch WT, Cook CB, et al. Clinical inertia. *Ann Intern Med*. 2001;135(9):825-834.
26. Grant R, Adams AS, Trinacty CM, et al. Relationship between patient medication adherence and subsequent clinical inertia in type 2 diabetes glycemic management. *Diabetes Care*. 2007;30(4):807-812.
27. Natali A, Ferrannini E. Effects of metformin and thiazolidinediones on suppression of hepatic glucose production and stimulation of glucose uptake in type 2 diabetes: a systematic review. *Diabetologia*. 2006;49(3):434-441.
28. Migoya EM, Miller J, Larson P. Sitagliptin, a selective DPP-4 inhibitor, and metformin have complementary effects to increase active GLP-1 concentrations. Oral presentation at: 67th Annual Meeting of the American Diabetes Association; June 2007; Chicago, IL; A74.
29. Vaag AA. Glycemic control and prevention of microvascular and macrovascular disease in the Steno 2 study. *Endocr Pract*. 2006;12(suppl 1):89-92.
30. U.K. Prospective Diabetes Study (UKPDS) Group. Tight blood pressure control and risk of macrovascular and microvascular complications in type 2 diabetes: UKPDS 38. *BMJ*. 1998;317(7160):703-713.
31. Misra A, Kumar S, Vikram NK, Kumar A. The role of lipids in the development of diabetic microvascular complications: implications for therapy. *Am J Cardiovasc Drugs*. 2003;3(5):325-338.
32. Caballero AE, Delgado A, Aguilar-Salinas CA, et al. The differential effects of metformin on markers of endothelial activation and inflammation in subjects with impaired glucose tolerance: a placebo-controlled, randomized clinical trial. *J Clin Endocrinol Metab*. 2004;89(8):3943-3948.
33. Jager A, van Hinsbergh VWM, Kostense PJ, et al. Von Willebrand factor, C-reactive protein, and 5-year mortality in diabetic and non-diabetic subjects: The Hoorn Study. *Arterioscler Thromb Vasc Biol*. 1999;19(12):3071-3078.
34. Jager A, van Hinsbergh VWM, Kostense PJ, et al. Increased levels of soluble vascular cell adhesion molecule 1 are associated with risk of cardiovascular mortality in type 2 diabetes: the Hoorn study. *Diabetes*. 2000;49(3):485-491.
35. Danesh J, Wheeler JG, Hieshfield GM, et al. C-reactive protein and other circulating markers of inflammation in the prediction of coronary heart disease. *N Engl J Med*. 2004;350(14):1387-1397.
36. Tanaka Y, Uchino H, Shimizu T, et al. Effect of metformin on advanced glycation endproduct formation and peripheral nerve function in streptozotocin-induced diabetic rats. *Eur J Pharmacol*. 1999;376(1-2):17-22.
37. Fruehwald-Schultes B, Oltmanns KM, Toschek B, et al. Short-term treatment with metformin decreases serum leptin concentration without affecting body weight and body fat content in normal-weight healthy men. *Metabolism*. 2002;51(4):531-536.
38. Krishnan STM, Baker NR, Carrington AL, Raymann G. Comparative roles of microvascular and nerve function in foot ulceration in type 2 diabetes. *Diabetes Care*. 2004;27(6):1343-1348.
39. Murphie P. Macrovascular disease aetiology and diabetic foot ulceration. *J Wound Care*. 2001;10(4):103-107.

©2009 American Medical Association. All rights reserved.
