# Supplementary material for: Metformin and N-terminal pro B-type natriuretic peptide in type 2 diabetes patients, a post-hoc analysis of a randomized controlled trial
Source: PLoS One. 2021 Apr 8;16(4):e0247939. doi: 10.1371/journal.pone.0247939 (PMC8031400; doi:10.1371/journal.pone.0247939)
Supplement: S3 File — (PDF) [file pone.0247939.s004.pdf]

THE VALUE OF METFORMIN
IN TYPE 2 DIABETES REQUIRING INSULIN THERAPY.

A treatment strategy for insulin resistance
in type 2 diabetes mellitus.

A. Kooy et al.

1. Introduction.

Type 2 diabetes mellitus (type 2 DM) is the most common hyperglycaemic state, and exists in all populations with a prevalence varying between 1 and 40%, being in whites about 2 % according to the modern criteria for the definition of diabetes [1].

Type 2 DM is a heterogeneous disorder with defects in insulin secretion as well as insulin action [1-3]. When type 2 DM is diagnosed, both insulin resistance and impaired betacell function exist: the secretion of insulin is insufficient to overcome the prevailing insulin resistance [1-3]. Although a small decrease in insulin receptor number has been demonstrated in these patients [4], the major defect of insulin action is situated at the postreceptor level [5].

When treatment with diet and oral hypoglycaemic agents has failed in type 2 DM, insulin therapy is warranted [6]. However, it seems not logical to treat with insulin alone. The 'extra' high doses of insulin needed to overcome the prevailing insulin resistance may result in weight gain and hyperinsulinaemia being associated with an increased risk for the development of atherosclerosis, ischaemic heart disease, dyslipoproteinaemia and hypertension [7-10]. Insulin stimulates arterial smooth muscle cell proliferation, promotes lipid uptake and synthesis in the arterial wall [9], and possibly increases platelet aggregation [10].

Metformin is able to increase the sensitivity of peripheral tissue to insulin and may prevent or reduce hyperinsulinaemia [11-14]. The combination of insulin and metformin as a treatment strategy in type 2 DM has not been extensively studied before. Therefore, in the present study we will investigate prospectively the potentially favourable effects of this drug in type 2 DM requiring insulin therapy on the quality of the diabetes regulation, the daily insulin usage, the lipid profile, the blood pressure, the incidence/progression of microvascular and macrovascular complications and the quality of life (Diabetes Health Profile).

2. Drug information.

Metformin. Clinical experience has proved metformin to be safe, effective and of additional value in reducing plasma glucose concentrations in patients with type 2 DM [15,16]. It does not stimulate insulin secretion, which explains the very rare occurrence of hypoglycaemia [15-17], but increases the sensitivity of peripheral tissue to insulin [11-14].

The occurrence of lactic acidosis is extremely rare, especially if compared with the occurrence during phenformin therapy, since metformin does not inhibit glucose oxidation or alter lactate turnover [16-20]. Metformin is not metabolized, and excreted unchanged in the urine, with a plasma half-life of six hours [16,18].

Insulin. Intensive insulin therapy 4 times daily will be propagated and applied, as proposed in The Diabetes Control and Complications Trial [21] (group I and II (N_{I} = N_{II} = 150)). A minority of patiens will be treated by usual insulin "mixtures" (10-30% rapidly and 70-90% slowly acting) 2 times daily (group III and IV (N_{III} = N_{IV} = 50)).

3. Subjects, patients to be included.

Inclusion-criteria.

1. Type 2 DM requiring insulin therapy after failure of maximal oral antidiabetic treatment (glycosylated Hb > 7.5%) and need for exogenous insulin.
2. Absence of Islet Cell Antibodies.
3. Intensive insulin therapy 4 times daily [21], or insulin therapy 2 times daily using insulin "mixtures" (10-30% rapidly and 70-90% slowly acting).
4. Standard dietary prescription by the dietitian (prescription enclosed).
5. Age: 30-80 years (during enrollment period).
6. Informed consent.

Exclusion-criteria.

1. Lack of cooperation.
2. Metformin-induced lactic acidosis.
3. Intolerance for metformin.
4. Impaired renal function (creatinin clearance < 60 ml/min).
5. Liver disease with hepatic failure.
6. Females in lactation period or being/becoming pregnant within 4 years.
7. Congestive heart failure, NYHA-classes III and IV.

4. Study design.

After enrollment ***4 main groups of patients*** (I - IV) are studied in a double-blind, prospective setting and treated according to the following protocol:

I: insulin 4 times daily plus 3 dd placebo (n_I = 150).

II: insulin 4 times daily plus 3 dd metformin 850 mg (n_{II} = 150).

III: insulin ("mixture") 2 times daily plus 3 dd placebo (n_{III} = 50).

IV: insulin ("mixture") 2 times daily plus 3 dd metformin 850 mg (n_{IV} = 50).

Base-line characteristics of the patients are documented at the end of phase I (see below): age, sex, weight (Quetelet-index), smoking behaviour, blood pressure, duration of diabetes, type of insulin usage (2 or 4 times daily), duration of insulin therapy, family history of diabetes, initial fasting plasma glucose and glycosylated Hb, fasting plasma insulin and C-peptide, and fasting lipid-profile. From all patients 4 extra blood samples will be taken for additional serological parameters (endothelial markers, etc.). The patient groups will be matched for the Quetelet-index and sex by stratification.

The study is divided into ***four phases.***

Phase I. The pre-enrollment phase (3 months; insulin therapy).

Phase II. The enrollment and randomization phase (1 months; placebo or metformin titration upto 850 mg, from 1 to 3 times daily if tolerated).

Phase III. The short-term active treatment phase (3 months). In the metformin group, the patients take the maximal dose of metformin, 2550 mg daily, unless side effects dictate a reduction of the dose. Short-term effects of metformin are studied especially on daily insulin dosage, diabetes regulation, blood pressure, lipids and quality of life.

Phase IV. The long-term active treatment phase (4 years). Long term effects of metformin are studied especially on blood pressure, cardiovascular incidents, microvascular complications and quality of life. Two years after the start of the study, the endpoints will be evaluated in order to justify the need for continuing the study.

5. Aim and endpoints of the study.

This study will be performed to compare the following endpoints reached by the different treatment strategies:

1. The quality of the "diabetes regulation".

    Aims of treatment according to DCCT (target values):

    # fasting plasma glucose : 4-7 mmol/l

    # non-fasting plasma glucose : 4-10 mmol/l

    # glycosylated Hb : < 7.0 %

    # incidence and severity of hypoglycemia

2. The degree of (hyper)insulinaemia.

    # fasting plasma insulin

    # daily dosage of insulin

3. The occurrence of dyslipoproteinaemia.
4. The occurence of weight gain, hypertension and cardiovascular incidents.
5. The development and/or progression of retino-, nefro- and neuropathy.
6. The quality of life (Diabetes Health Profile).
7. Pharmaco-economic evaluation (Merck).

6. Treatment of hypertension and dyslipoproteinaemia.

Standardized protocols are used in the different active treatment phases (III and IV). During the (pre-) enrollment phases (I and II) the criteria of phase III are used for treatment.

6.1. Phase III. The short-term active treatment phase (3 months).

albuminuria > 1000 mg/24h:

enalapril upto 40 mg daily, if tolerated (initial blood pressure > 130/90 mm Hg).

LDL-cholesterol > 7.0 mmol/l, while gly-Hb < 8.0% :

(1) diet and recommendations according to the AHA-protocol

(AHA = American Heart Association)

(2) simvastatin upto a maximal daily dosage of 40 mg a.n.

(3) simvastatin 40 mg combined with acipimox 250 mg, upto 3 times daily

triglycerids > 5.0 mmol/l, while Gly-Hb < 8.0 % :

(1) diet and recommendations according to the AHA-protocol

(2) acipimox 250 mg, upto 3 times daily

HDL < 1.0: not treated

(**6.1. Phase III** continued)

hypertension > 180/110 mm Hg: treatment according to the following algorythm:

1. First choice:	1.1.	enalapril upto 40 mg daily, if tolerated
	1.2.	enalapril 40 mg combined with hydrochloorthiazide upto 25 mg daily, if tolerated
2. Second choice:	2.1.	kaliumlosartan upto 100 mg daily, if tolerated
	2.2.	kaliumlosartan 100 mg combined with hydrochloorthiazide upto 25 mg daily, if tolerated

3. If the response to the first or second choice is insufficient (blood pressure still > 180/110 mm Hg), additional treatment with nifedipine upto 60 mg daily will be given.

6.2. Phase IV. The long-term active treatment phase (4 years).

The treatment of the lipid profile during this phase is in accordance with the strict criteria as recommended by the American Heart Association.

microalbuminuria > 30 mg/24h:

enalapril upto 40 mg daily, if tolerated (initial blood pressure > 130/90 mm Hg).

LDL-cholesterol > 2,6 mmol/l, while gly-Hb < 8.0% :

(1) diet and recommendations according to the AHA-protocol

(2) simvastatin 20 mg upto a maximal daily dosage of 80 mg a.n.

(3) simvastatin 80 mg combined with acipimox 250 mg, upto 3 times daily

triglycerids > 2.3 mmol/l, while gly-Hb < 8.0% :

(1) diet and recommendations according to the AHA-protocol

(2) acipimox 250 mg, upto 3 times daily

HDL < 0.9: acipimox 250 mg, upto 3 times daily

hypertension > 150/90 mm Hg: treatment according to the following algorythm:

1. First choice:	1.1.	enalapril upto 40 mg daily, if tolerated
	1.2.	enalapril 40 mg combined with hydrochloorthiazide upto 25 mg daily, if tolerated
2. Second choice:	2.1.	kaliumlosartan upto 100 mg daily, if tolerated
	2.2.	kaliumlosartan 100 mg combined with hydrochloorthiazide upto 25 mg daily, if tolerated

3. If the response to the first or second choice is insufficient (blood pressure still > 150/90 mm Hg), additional treatment with nifedipine upto 60 mg daily will be given.

7. Parameters to be studied.

Several parameters will be studied with different intervals.

7.1. Parameters taken on a 2-week-basis:

* Values of plasma glucoses (3 times daily before the meals and once ante noctem, corresponding with values taken at approximately 7.00-12.00-17.00-22.00 h; $\mu \pm$ sd). All these values as well as the hypoglycaemia's and possible adverse events will be documented in a diary.
* Documentation of co-medication.

7.2. Parameters taken on a 3-month-basis:

7.2.1. Standard clinical history.

physical exercise	WONCA-scoring system		
dietary problems	scoring system (dietitian)		
palpitations	-/+		
micturational problems	-/+		
impotence	-/+		
loss of libido	-/+		
orthostasis	-/+		
nausea/vomitus	-/+		
nocturnal diarrhoea	-/+		
hypoglycaemia	scoring system		
paraesthesia	-/+		
passagère pareses	-/+		
skin lesions	-/+		
alcohol usage	U/day:		
nicotine usage	U/day:		
**cardiovascular incidents:**			
angina pectoris	NYHA-scoring system, eventually completed with coronary angiographical findings		
coronary intervention	PTCA	-/+	date:
	CABG	-/+	date:
myocardial infarction		-/+	date:

TIA		-/+	date:
CVA		-/+	date:
necrotectomy		-/+	date:
amputation		-/+	date:
dialysis	hemodialysis	-/+	starting date:
	CAPD	-/+	starting date:
kidney transplantation		-/+	date:

7.2.2. Standard physical examination.

length, weight, BMI

blood pressure, orthostasis, heart rate

souffles	carotis	R -/+	L -/+
	femoralis	R -/+	L -/+
pulsations	femoralis	R -/+	L -/+
	tib post	R -/+	L -/+
	dors ped	R -/+	L -/+
reflexes	KTR	R N/↓/↑	L N/↓/↑
	ATR	R N/↓/↑	L N/↓/↑
	FSR	R N/↓/↑	L N/↓/↑

sensibility intact tested by monofilament	-/+
sense of vibration intact	-/+
lesions in injection areas	-/+
lesions on feet	-/+

7.2.3. Laboratory findings.

* fasting plasma values of gly-Hb, Hb, reticulocytes, creatinin, electrolytes.

7.3. Parameters taken at baseline, at the end of phase III, and subsequently on a half-year-basis:

* dietary history by dietition (protocol).
* fasting plasma values of insulin, C-peptide, anti-insulin antibodies, venous bloodgas analysis (Astrup), lipid-profile (cholesterol, HDL, LDL, TG), lactate and metformin [22]; 4 extra blood samples for additional serological parameters (endothelial markers, etc.).
* electrocardiogram.
* albuminuria (overnight-sample and albuminuria/24h).

7.4. Parameters taken on a one-year-basis:

* fundoscopy by ophthalmologist (protocol).
* ambulant registration of blood pressure during 24 hours.

8. Literature.

1. Unger RH and Foster DW. Diabetes Mellitus. In: Williams' Textbook of Endocrinology, 8th ed.; pp.: 1255-1333; Wilson JD & Foster DW, eds., Philadelphia 1992.
2. DeFronzo RA. The triumvirate beta cell, muscle, liver: a collusion responsible for NIDDM. Diabetes 1988;37:667-687.
3. Robertson RP. Type II diabetes, glucose "non-sense", and islet desensitization. Diabetes 1989;38:1501-5.
4. Olefsky JM. The insulin receptor: its role in insulin resistance in obesity and diabetes. Diabetes 1976;25:1154-65.
5. Rizza RA, Mandarino LJ, Gerich JE. Mechanism and significance of insulin resistance in non-insulin-dependent diabetes mellitus. Diabetes 1981;30:990-5.
6. Heine RJ. Insulin treatment of non-insulin-dependent diabetes mellitus. In: Baillière's Clinical Endocrinology and Metabolism, Non-insulin-dependent diabetes. Nattrass M, Hale PJ, eds., London, 1988.
7. Stout RW. Insulin and atheroma - a 20-year perspective. Diabetes care 1990;631-54.
8. Jarrett RJ. Is insulin atherogenic ? Diabetologia 1988;31:71-5.
9. Stolar MW. Atherosclerosis in diabetes: the role of hyperinsulinemia. Metabolism 1988;37 (suppl.1):1-9.
10. Robertson DA, Hale PJ, Nattrass M. Macrovascular disease and hyperinsulinaemia. In: Baillière's Clinical Endocrinology and Metabolism, Non-insulin-dependent diabetes. Nattrass M, Hale PJ, eds., London, 1988.
11. Nosadini R, Avogaro A, Trevisian R, et al. Effect of metformin on insulin-stimulated glucose turnover and insulin binding to receptors in type II diabetes. Diabetes Care 1987;10:62-7.
12. Johnson AB, Webster JM, Sum C-F, et al. The impact of metformin therapy on hepatic glucose production and skeletal muscle glycogen synthase activity in overweight type II diabetic patients. Metabolism 1993;42:1217-22.

13. Hother-Nielsen O, Schmitz O, Andersen PH, Beck-Nielsen H, Petersen O. Metformin improves peripheral but not hepatic insulin action in obese patients with type II diabetes. Acta Endocrinol 1989;120:257-65.

14 Klip A, Leiter LA. Cellular mechanism of action of metformin. Diabetes Care 1990;13: 696-704.

15. Bailey CJ. Biguanides and NIDDM. Diabetes Care 1992;15:755-72.

16. DeFronzo RA, Goodman AM, and the multicenter metformin study group. Efficacy of metformin in patients with non-insulin-dependent diabetes mellitus. N Engl J Med 1995; 333:541-9.

17. Reaven GM, Johnston P, Hollenbeck CB, et al. Combined metformin-sulfonylurea treatment of patients with noninsulin-dependent diabetes in fair to poor glycemic control. J Clin Endocrinol Metab 1992;74:1020-6.

18. Stumvoll M, Nurjhan N, Perriello G, Dailey G, Gerich JE. Metabolic effects of metformin in non-insulin-dependent diabetes mellitus. N Engl J Med 1995;333:550-4.

19. Crofford OB. Metformin (Editorial). N Engl J Med 1995; 333:588-9.

20. Cusi K, Consoli K. Effect of metformin on glucose and lactate metabolism in NIDDM. Diabetes 1994;43:Suppl 1: 258A, abstract.

21. The Diabetes Control and Complications Trial Research Group. The effect of intensive treatment of diabetes on the development and progression of long-term complications in insulin-dependent diabetes mellitus. N Engl J Med 1993;329:977-86.

22. Charles BG, Jacobson NW, Ravenscroft PJ. Rapid liquid-chromatograpgic determination of metformin in plasma and urine. Clin Chem 1981;27:434-6.

P.S. : Dhr. M.G. Wulffelé is als arts-onderzoeker per 1 oktober aan dit project verbonden.

Adriaan Kooy, MD PhD
Internist of Vascular Medicine
Director Bethesda Diabetes RC
Associate Professor UMCG
Hoogeveen, Groningen, NL
+31 (0)528 286859
